# Development of a high-throughput UHPLC–MS/MS multi-method for the analysis of mycotoxins in plant-based milk alternatives as the basis for risk assessment

**DOI:** 10.3389/fnut.2025.1709925

**Published:** 2026-01-15

**Authors:** Fabian Dick, Maximilian Schuster, Sarah Schneidemann-Bostelmann, Stefan Asam, Michael Rychlik

**Affiliations:** Chair of Analytical Food Chemistry, Technical University of Munich, Munich, Germany

**Keywords:** *Alternaria*, *Aspergillus*, *Fusarium*, LC-MS/MS analysis, modified mycotoxins, multi-mycotoxin analysis, mycotoxins, plant-based milk alternatives

## Abstract

Plant-based alternatives to animal-based foods represent a rapidly growing market, as more and more consumers are willing to include these products into their daily diets. One particular segment is plant-based milk alternatives (PBMAs). Environmental and ethical concerns mainly drive this growing trend. However, the raw materials used in the production of PBMAs are susceptible to mycotoxin contamination, and currently, there are no regulatory limits for mycotoxins in PBMAs. Therefore, analyzing PBMAs to get more information about the mycotoxin contamination in these products is of great interest. To obtain more exposure data, we developed a QuEChERS (quick, easy, cheap, effective, rugged, and safe)-based multi-mycotoxin method to analyze 31 *Alternaria*, *Aspergillus,* and *Fusarium* toxins in PBMAs using ultra-high-performance liquid chromatography–tandem mass spectrometry (UHPLC–MS/MS). The method was successfully validated and applied to 100 different PBMAs from oats, rice, spelt, almonds, hazelnuts, walnuts, cashews, soy, peas, and hemp. Our results showed a frequent contribution of PBMAs to the overall mycotoxin exposure, and some of these products have to be considered critical for toddlers from a risk-assessment perspective. In particular, PBMAs made from oats, almonds, hazelnuts, and tiger nuts were assessed as a risk for this age group.

## Highlights

- A stable isotope dilution assay for *Alternaria, Aspergillus,* and *Fusarium* toxins, including modified mycotoxins, was developed and validated.
- Sample clean-up was performed using a modified QuEChERS approach, and detection and quantification were carried out via UHPLC–MS/MS.
- Analysis of 100 plant-based milk alternatives (PBMAs) revealed frequent contamination with mycotoxins, corresponding to the raw material
- A comprehensive risk assessment of the analyzed PBMAs highlights the need to update the respective mycotoxin regulations and implement more rigorous control measures.

## Introduction

Plant-based alternatives are taking over steadily growing market shares from foods of animal origin. A particular segment is plant-based milk alternatives (PBMAs), which is expected to grow constantly by 10% per year (1). This increasing trend is mainly driven by consumers’ preference over conventional dairy products, which are often criticized concerning the welfare of farmed animals, the environmental impact on climate and biodiversity (2), and human health compromised by antibiotic resistance, among others.

The currently high variety of plant sources used for PBMAs historically started with “soy milk” in China or “Horchata” in Spain, the latter made from tiger nuts (3). Today, PBMAs produced from a broad range of cereals, pseudocereals, nuts, legumes other than soy, and even seeds such as hemp have found their way on the market.

Apart from the above-mentioned individual considerations for omitting animal-derived products, the impact of replacing bovine milk with PBMAs on human nutrition and food safety must also be considered. While the nutritional aspects regarding micronutrient content, protein content, and quality have been discussed elsewhere (4), the assessments of PBMA safety are relatively scarce, although plant materials for food use are known to contain a significant variety of contaminants. One of the rare investigations in this field focused on pesticides, heavy metals, microbiology, and mycotoxins in drinks made from soy, oats, and almonds (5).

**Figure 1:**
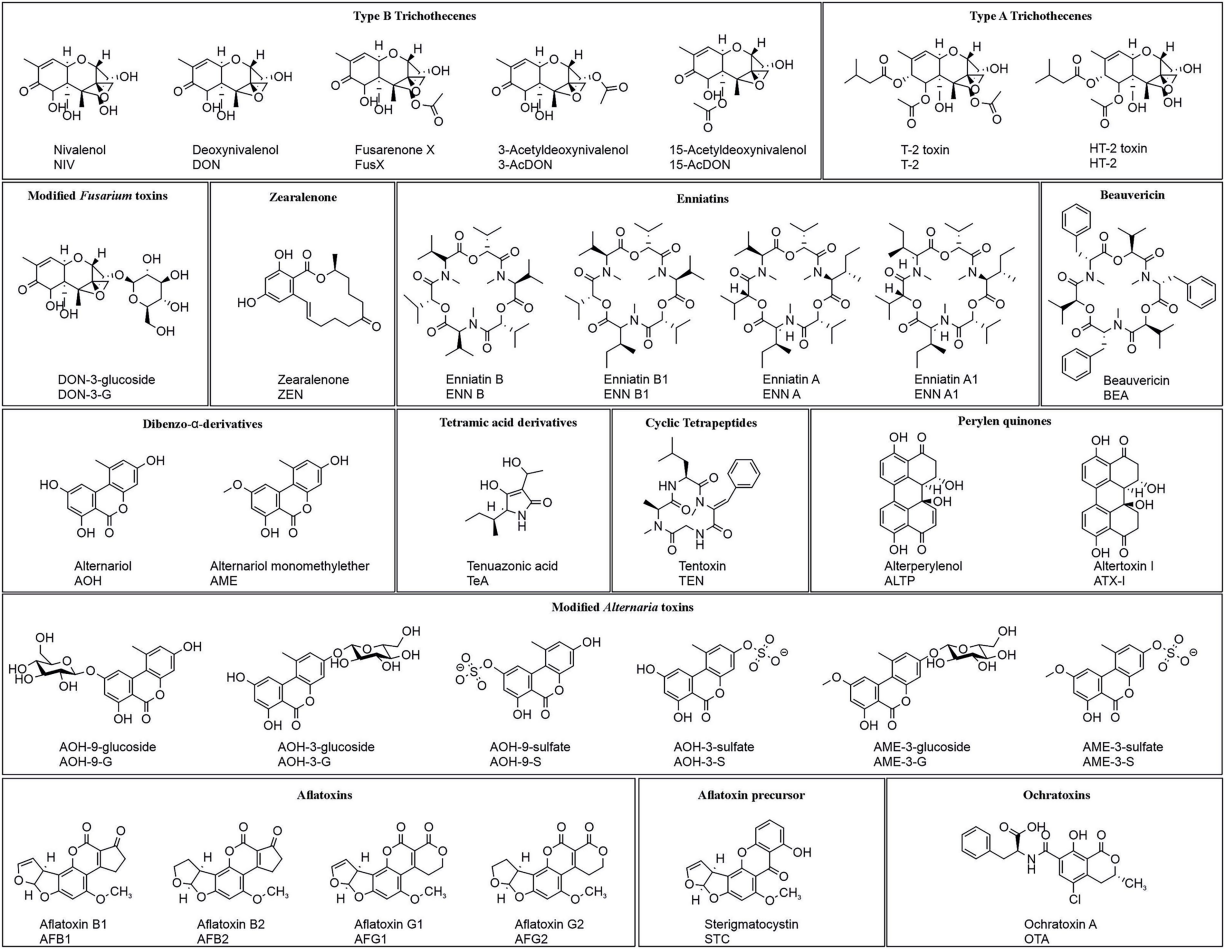
Structures of the 31 analyzed mycotoxins grouped by their molecular structure. 15-AcDON was not analyzed and is depicted here for the sake of completeness.

One particular group of contaminants of plant-based foods is mycotoxins, which are biosynthesized by fungi that frequently infect plant sources. Most of these fungi belong to the genera *Alternaria*, *Aspergillus*, *Fusarium,* and *Penicillium* (6), producing a wide range of different toxic metabolites (for structures, see Figure 1). Apart from this huge chemical diversity, previous studies have shown that the frequent co-occurrence of mycotoxins may lead to synergistic, antagonistic, or additive effects (7).

Mycotoxins can be classified based on their structural features, toxicity, origin, occurrence, or regulation. For our study, we grouped them into three categories: regulated, emerging, and modified mycotoxins. For the first group, regulatory limits for food commodities in many parts of the world have been established. The European Commission (EC) has collected maximum levels (ML) in Commission Regulation (EU) 2023/915 (8) for deoxynivalenol (DON), fumonisins, ochratoxin A (OTA), aflatoxin B1 (AFB1), aflatoxin B2 (AFB2), aflatoxin G1 (AFG1), aflatoxin G2 (AFG2) zearalenone (ZEN), and the type A trichothecenes T-2 toxin (T-2) and HT-2 toxin (HT-2), for the latter two of which MLs were recently introduced (9).

The second group, the emerging mycotoxins, referred to as “mycotoxins, which are neither routinely determined nor legislatively regulated” (10, 11), are toxins like the enniatins A, A1, B, and B1 (ENN A, ENN A1, ENN B, and ENN B1), beauvericin (BEA), nivalenol (NIV), fusarenone X (Fus X), sterigmatocystin (STC), tentoxin (TEN), alterperylenol (ALTP), altertoxin I (ATX I), and many others. For some of these, e.g., alternariol (AOH) or alternariol methyl ether (AME), guidance or indicative levels (ILs) have been introduced. If these are exceeded, the sources of the contamination must be elucidated and minimized (12).

The third group comprises the modified mycotoxins (13). In plant metabolism, conjugates of mycotoxins with glucose play the major role, whereas sulfates or acetates may originate from fungal biosynthesis of mycotoxins. Due to their frequent occurrence and potential toxicity, we included DON-3-glucoside (DON-3-G), AOH-3-glucoside (AOH-3-G), AOH-9-glucoside (AOH-9-G), AOH-3-sulfate (AOH-3-S), AOH-9-sulfate (AOH-9-S), AME-3-glucoside (AME-3-G), and AME-3-sulfate (AME-3-S) in our analytical method reported recently for cereals (14).

For the plant sources, from which the PBMAs are produced, a frequent co-occurrence of mycotoxins has been reported by Varga et al. (15) and may confer risk from their consumption. Moreover, a recent investigation reported on the contamination of drinks made from soy, almonds, and oats with the mycotoxins AFB1, STC, ZEN, DON, T-2, and HT-2 (5). Therefore, multi-mycotoxin methods must cover the compounds regulated in the respective drink or its sources and the toxins that can occur in any plant source, including emerging and modified toxins.

For quantifying mycotoxins, ultra-high-performance liquid chromatography coupled with tandem mass spectrometry (UHPLC–MS/MS) using stable isotope dilution analysis (SIDA) is considered the state of the art. However, analyzing many mycotoxins with a broad range of physicochemical polarities bears additional challenges. For an optimal yield of the analytes, extraction with solvents will necessarily lead to co-extraction of a high amount of matrix compounds, compromising the sensitivity of UHPLC–MS/MS due to matrix interferences. Dilute-and-shoot methods, on the one hand, would require high dilution factors and lead to low signal intensities. On the other hand, immunoaffinity columns (IACs) would not show enough cross-reactivity to bind all analytes. Therefore, we have already successfully applied a combination of QuEChERS (quick, easy, cheap, rugged, and safe) and dispersive solid-phase extraction (d-SPE) for the simultaneous quantitation of *Alternaria* and *Fusarium* toxins in cereals (14). The method was already capable of accurately and sensitively quantifying a wide variety of analytes, ranging from the most polar tenuazonic acid (TeA) to the non-polar ENNs and AME. We have also recently developed a UHPLC–MS/MS multi-method for the analysis of mycotoxins in plant-based meat, cheese, and fish alternatives, covering 15 mycotoxins, and performed some risk assessments for these products (16).

Therefore, this study’s first aim was to expand our existing methods to include additional mycotoxins and to modify the workup for liquid samples. After method development and validation, the second aim was to apply the technique to a wide range of PBMAs to evaluate the risk for consumers related to the consumption of these products.

## Materials and methods

### Reagents and chemicals

Water (LC–MS grade and HPLC grade) and acetonitrile (ACN) (HPLC grade) were purchased from Th. Geyer (Renningen, Germany), methanol (MeOH) (LC–MS grade), and ACN (LC–MS grade) were obtained from Honeywell Riedel-de Haen (Seelze, Germany). Sodium chloride and ammonia solution (NH_4_OH) were purchased from VWR (Ismaning, Germany) in analytical or purer grade. Anhydrous magnesium sulfate, ammonium formate (NH_4_HCO_2_), and formic acid (FA) were provided by Sigma Aldrich (Steinheim, Germany) in analytical grade. The readily prepared d-SPE tubes Supel™ QuE PSA/C18 tube (150 mg Supelclean™ PSA, 150 mg Discovery™ C18, 900 mg magnesium sulfate), and d-SPE tubes Supel™ QuE PSA tube (150 mg Supelclean™ PSA, 900 mg magnesium sulfate), were bought from Sigma Aldrich (Steinheim, Germany).

### Analytical standards

Reference standards for AOH, AME, ALTP, ATX I, AOH-3-G, AOH-9-G, AOH-3-S, AOH-9-S, AME-3-G, AME-3-S were either isolated from fungal extracts or synthesized as described in the literature (17).

The other reference compounds were obtained commercially from the respective sources indicated in brackets: TEN and TeA (Merck, Darmstadt, Germany); DON, 3-AcDON, Fus X (Coring System Diagnostix, Gernsheim, Germany), T-2 (LGC Standards/Dr. Ehrenstorfer, Wesel, Germany), BEA, HT-2, and ZEN (Sigma Aldrich, Steinheim, Germany), NIV, ENN A and ENN B (Cayman Chemicals, Michigan, United States), ENN A1 and ENN B1 (Enzo Life Science, New South Wales, Australia), STC, OTA, AFB1, AFB2, AFG1, and AFG2 (Biopure, Tulln, Austria).

The stable isotope-labeled standards (ILS) [^2^H_4_]-AOH, [^2^H_4_]-AME, [^13^C_6_]-TeA, [^15^N_3_]-ENN A1, and [^4^C_13_]-T-2 were synthesized as reported previously (18–21). [^13^C_15_]-DON, [^13^C_17_-3-AcDON], [^13^C_20_]-OTA, [^13^C_18_]-STC, [^13^C_17_]-AFB1, [^13^C_17_]-AFB2, [^13^C_17_]-AFG1, [^13^C_17_]-AFG2 were obtained from Libios (Vindry Sur Turdine, France), and DON-3-G, [^13^C_21_]-DON-3-G and [^13^C_22_]-HT-2 were purchased from Biopure (Tulln, Austria).

### Preparation of stock solutions

All reference compounds were quantified by quantitative nuclear magnetic resonance (qNMR) measurements as described in the literature (22). Stock solutions were prepared in ACN at concentrations ranging from 0.0001 to 100 μg/mL. The stability of the standards was assessed as reported in the literature (17). Accordingly, quality control standards were employed, and their concentrations were monitored through long-term observation of signal intensities. The standards were measured regularly to ensure the right concentration and instrument performance. The standards were stored at −20 °C between these measurements until further use.

### Method development

For workup optimization, PBMAs were spiked in duplicates with the respective toxins. Our previously developed method (14), along with QuEChERS approaches described in the literature (23), served as a blueprint for the procedure.

### Extraction and sample volume

Sample volume, extraction time, and extraction volume were tested in various combinations. Different proportions of ACN and water, and adding FA, were tested for the extraction solvent. To achieve high sensitivity, two sample volumes—5 and 10 mL— were tested. The ratio of sample volume to ACN varied between the ratios 2:1, 1:1, and 1:2. The FA content varied between 0, 0.5, 1, and 2% FA. Extraction time was tested for 0, 10, 20, 30, 60, and 120 min shaking at 350 min^−1^ using a horizontal shaker (Kombischüttler KL 2, Edmund Bühler GmbH, Hechingen, Germany), with 0 min involving just strong manual shaking for 30 s.

### QuEChERS clean-up

The composition of the QuEChERS salts was based on the originally described QuEChERS approach (23). QuEChERS clean-up was performed using 4 g MgSO_4_ and 1 g NaCl for all sample-to-solvent ratios.

### Defatting

As the analyzed products contained lipids, we tested a defatting step after the extraction step using 0, 1, 2, and 5 mL of cyclohexane. The cyclohexane was added to the supernatant after the QuEChERS step and was shaken for 5 min. After that, the upper cyclohexane phase was discarded, and the ACN phase was used for further clean-up.

### d-SPE clean-up

Based on our previous experiments, we tested the most promising d-SPE combinations, namely PSA (150 mg Supelclean™ PSA, 900 mg magnesium sulfate) and a combination of PSA and C18 (150 mg Supelclean™ PSA, 150 mg Discovery™ C18, 900 mg magnesium sulfate). Both types of d-SPE tubes were purchased from Sigma Aldrich (Steinheim, Germany). Ten milliliters of the ACN layer was transferred into centrifuge tubes containing the d-SPE sorbents. After shaking the d-SPE tubes for 15 min, the tubes were centrifuged, and 8 mL of the supernatant was collected for drying. The shaking time varied between 0, 5, 10, 15, 30, and 60 min.

### Reconstitution solvent

The following volumetric ratios of MeOH/H_2_O were tested for reconstitution of the dried sample extract: 7:3, 6:4, 5:5, 4:6, and 3:7.

### Final method

Ten milliliters of the PBMA samples were pipetted into a 50-mL centrifuge tube. The sample was spiked with the ILS; details on the respective amounts are provided in Supplementary Table 1. After short manual shaking and vortexing the sample, 11 mL of ACN (LC–MS grade) containing 1% FA was added to the sample, and the extraction tube was vortexed and shaken for 10 min at 350 min^−1^ on a horizontal shaker (Kombischüttler KL 2, Edmund Bühler GmbH, Hechingen, Germany). Following centrifugation (Centrifuge 5,810 R, Eppendorf AG, Hamburg, Germany) for 5 min at 3,220 g, the supernatant was transferred to the d-SPE tube.

The supernatant was mixed with 4 g MgSO_4_ and 1 g NaCl in a 50-mL centrifuge tube for a QuEChERS clean-up. The tubes were shaken vigorously by hand for 60 s and then centrifuged at 3220 g for 10 min.

A total of 9.5 mL of the upper ACN phase was added to the d-SPE tube (150 mg Supelclean™ PSA, Discovery™ C18, 900 mg magnesium sulfate). The tube was shaken vigorously by hand for 30 s and then shaken on a horizontal shaker at 350 min^−1^ for 15 min. The d-SPE tube was centrifuged at 3,220 g for 15 min. After centrifugation, 8.5 mL of the supernatant was transferred to a 15-mL centrifuge tube.

Four milliliters of the supernatant was transferred to a 4-mL vial and dried at 40 °C under a constant nitrogen stream (Evaporator EVA-EC2-48-S, Zefa-Laborservice, Harthausen). When the vial was almost empty, it was refilled with an additional 4 mL of supernatant and dried completely. The dried residue was reconstituted in 200 μL MeOH/H_2_O (6/4, v/v), transferred to 1.5 mL plastic microtubes, and frozen at −20 °C for at least 30 min. The frozen microtubes were centrifuged (Laborzentrifuge 2 K15, Sigma, Osterode am Harz, Germany) for 15 min at 13,201 g at 4 °C. The supernatant was membrane-filtered (PVDF, 0.2 μm) into 1.5-mL glass vials containing micro-inserts. Samples were stored at −18 °C until analysis.

The work-up was conducted in duplicates. If the relative standard deviation (RSD) of any measurement was above 20% for analytes quantified via SIDA and 40% for analytes quantified via matrix-matched calibration (MMC), the work-up was repeated. However, the RSD was below 15% in most first work-ups.

### LC–MS/MS analysis

**Figure 2:**
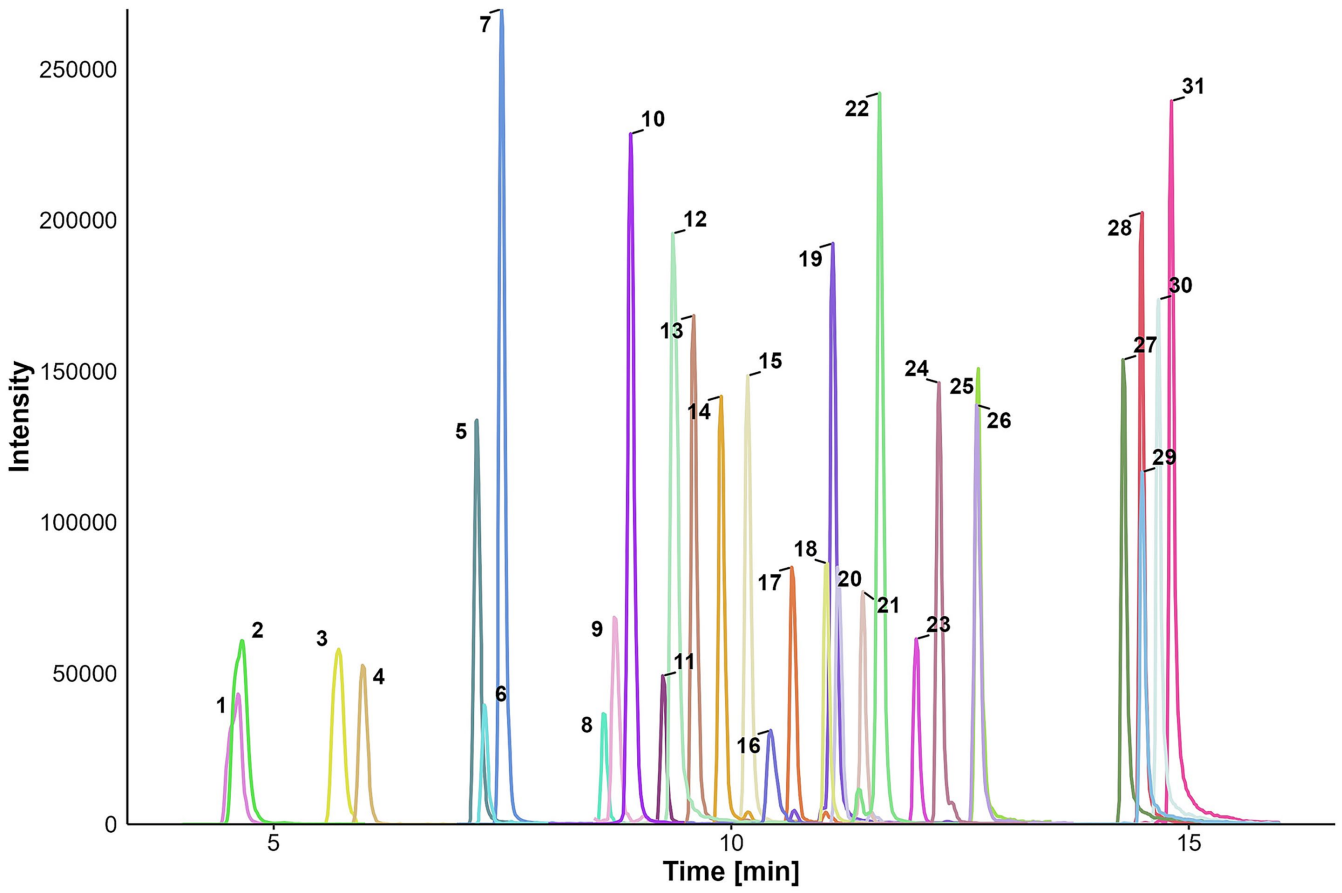
Chromatogram of all 31 analytes. (1) NIV, (2) TeA, (3) DON, (4) DON-3-G, (5) AOH-3-G, (6) Fus X, (7) AOH-3-S, (8) 3-AcDON, (9) AOH-9-S, (10) AOH-9-G, (11) AFG2, (12) AOH, (13) AFG1, (14) AFB2, (15) AFB1, (16) OTA, (17) AME-3-S, (18) ATX I, (19) ALTP, (20) AME-3-G, (21) HT-2, (22) TEN, (23) T-2, (24) ZEN, (25) AME, (26) STC, (27) ENN B, (28) BEA, (29) ENN B1, (30) ENN A1, (31) ENN A.

For the LC–MS/MS method, we employed our previously developed method (14) and included the *Aspergillus* toxins AFB1, AFB2, AFG1, AFG2, OTA, and STC. The addition of the transitions in the existing method worked well and did not require any changes in the solvent, gradient, or source conditions. The chromatogram of all analytes is shown in Figure 2.

### LC parameters

A Shimadzu Nexera X2 UHPLC system (Shimadzu, Kyoto, Japan) was used for liquid chromatography. Chromatographic separation for all toxins was achieved on a Waters BEH C18 UHPLC column (Acquity BEH C18, 100 mm, 1.7 μm × 2.1 mm; Waters GmbH, Eschborn, Germany). The column was kept at 40 °C, and a 0.3 mL/min flow rate was used. An aqueous NH_4_HCO_2_ solution (5 mmol/L) at pH 9 was used for mobile phase A, and pure MeOH for mobile phase B. The pH was adjusted using 25% NH_4_OH. For better sensitivity, an injection volume of 10 μL was used. For obtaining a better peak shape for early eluting analytes, the co-injection of 40 μL H_2_O with NH_4_HCO_2_ (5 mmol/L) at pH 9 was used (20 μL before and after the 10 μL sample, respectively). The binary gradient was programmed as follows: 0–2 min, 5% B; then increased to 18% B in 1 min. A slow increase was applied within 2 min to 25% B. The concentration of B was then raised to 90% in 8 min and further raised to 99% B in 0.5 min. Finally, 99% B was held for 2 min. The concentration of solvent B was then brought back to 5% over 3.5 min, followed by a 5-min equilibration.

### MS parameters

The UHPLC system was coupled to a Shimadzu 8,050 triple quadrupole mass spectrometer (Shimadzu Corporation, Kyoto, Japan) with an electrospray ionization (ESI) source. The following ion source parameters were used: heat block temperature, 450 °C; interface temperature, 350 °C; desolvation temperature, 150 °C; interface voltage; 3 kV for positive ionization and −3 kV for negative ionization; drying gas flow, 10 L/min; heating gas flow, 10 L/min; nebulizing gas flow, 3 L/min; and collision-induced dissociation gas pressure, 270 kPa. Polarity switching enabled simultaneous measurement in positive and negative ESI mode in the same LC–MS/MS run. Different ionization polarities were necessary for increased analyte sensitivity. All measurements were conducted in multiple reaction monitoring (MRM) mode. All mass transitions used in the final method are shown in Table 1.

**Table 1:** MS/MS parameters for all mycotoxins and their internal standards in alphabetical order.

Analyte	Measured species	Precursor ion [m/z]	Product ion [m/z]	Q1 prebias [V]	CE [V]	Q3 prebias [V]	Rt [min]
3-AcDON	[M + H]^+^	339.1	231.3/175.2	16/16	13/25	26/20	8.73
[^13^C_17_]-3-AcDON	[M + H]^+^	356.1	245.3/186.2	16/16	13/25	26/20	8.73
AFB1	[M + H]^+^	313.0	285.2/241.1	−16/−16	−23/−36	−20/−26	10.33
[^13^C_17_]-AFB1	[M + H]^+^	330.0	301.2/256.1	−16/−16	−23/−36	−20/−26	10.33
AFB2	[M + H]^+^	315.0	287.2/259.1	−16/−16	−26/−30	−20/−18	10.03
[^13^C_17_]- AFB2	[M + H]^+^	332.0	303.2/274.1	−16/−16	−26/−30	−20/−18	10.03
AFG1	[M + H]^+^	329.0	243.2/200.1	−16/−14	−26/−43	−26/−22	9.72
[^13^C_17_]-AFG1	[M + H]^+^	346.0	257.2/212.1	−16/−14	−26/−43	−26/−22	9.72
AFG2	[M + H]^+^	331.2	189.1/115.2	−18/−14	−42/−65	−20/−24	9.39
[^13^C_17_]- AFG2	[M + H]^+^	348.2	330.2/123.2	−18/−14	−42/−65	−20/−24	9.39
ALTP	[M – H]^−^	349.2	303.1/261.0	12/12	19/29	28/26	11.20
AOH	[M – H]^−^	257.3	213.0/214.9	28/26	25/23	34/14	9.45
[^2^H_4_]-AOH	[M – H]^−^	261.3	217.0/218.9	28/26	25/23	34/14	9.45
AOH-3-G	[M – H]^−^	419.1	256.2/255.1	30/16	33/44	26/26	7.22
AOH-9-G	[M – H]^−^	419.1	256.2/255.1	30/16	33/44	26/26	8.82
AOH-3-S	[M – H]^−^	337.1	257.2/213.1	24/24	22/40	24/18	7.49
AOH-9-S	[M – H]^−^	337.1	257.2/213.1	24/24	22/40	24/18	8.77
AME	[M – H]^−^	271.3	256.0/255.1	14/34	23/28	10/26	12.76
[^2^H_4_]-AME	[M – H]^−^	275.3	260.0/259.1	14/34	23/28	10/26	12.76
AME-3-G	[M – H]^−^	433.3	270.2/271.2	16/12	33/26	18/20	11.20
AME-3-S	[M – H]^−^	351.2	271.2/256.2	12/12	23/35	26/24	10.84
ATX I	[M – H]^−^	351.2	315.0/333.1	26/26	17/12	18/36	11.20
BEA	[M + NH_4_]^+^	801.5	244.2/134.2	−18/−18	−33/−55	−18/−14	14.50
DON	[M + H]^+^	297.2	249.2/231.2	−14/−18	−11/−12	−18/−26	5.75
[15C13]-DON	[M + H]^+^	312.2	263.2/245.2	−14/−18	−11/−12	−18/−26	5.75
DON-3-G	[M – H]^−^	457.3	427.1/255.4	22/16	17/27	44/30	6.05
[^13^C_21_]-DON-3-G	[M – H]^−^	478.2	447.1/261.3	22/16	17/27	44/30	6.05
ENN A1	[M + NH_4_]^+^	685.5	210.3/100.3	−20/−16	−29/−55	−14/−22	14.70
[^15^N_3_]-ENN A1	[M + NH_4_]^+^	688.5	211.3/101.3	−20/−16	−29/−55	−14/−22	14.70
ENN A	[M + NH_4_]^+^	699.5	210.2/100.3	−16/−16	−33/−55	−22/−24	14.85
ENN B1	[M + NH_4_]^+^	671.4	196.2/210.3	−16/−16	−36/−32	−14/−14	14.65
ENN B	[M + NH_4_]^+^	657.5	196.2/86.2	−18/−26	−32/−64	−14/−18	14.30
Fus X	[M + H]^+^	372.2	355.3/337.2	−18/−18	−9/−13	−18/−24	7.40
HT-2	[M + NH_4_]^+^	442.2	263.3/215.2	−22/−22	−14/−14	−10/−24	11.57
[^13^C_22_]-HT-2	[M + NH_4_]^+^	464.2	278.3/200.2	−22/−22	−14/−14	−10/−24	11.57
NIV	[M – H]^−^	311.2	281.2/191.2	22/12	11/21	16/22	4.65
OTA	[M – H]^−^	402.3	358.2/167.1	20/20	21/36	14/14	10.62
[^13^C_20_]-OTA	[M – H]^−^	422.3	377.2/174.1	20/20	21/36	14/14	10.62
STC	[M + H]^+^	324.9	310.2/281.2	−16/−16	−23/−36	−20/−26	12.81
[^13^C_18_]-STC	[M + H]^+^	342.9	327.2/298.2	−16/−16	−23/−36	−20/− 26	12.81
T-2	[M + NH_4_]^+^	484.4	305.2/215.3	−26/−28	−15/−21	−22/−24	12.13
[^13^C_4_]-T-2	[M + NH_4_]^+^	488.4	307.2/216.3	−26/−28	−15/−21	−22/−24	12.13
TeA	[M – H]^−^	196.4	111.9/139.0	22/22	25/19	34/26	4.65
[^13^C_6_, ^15^N]-TeA	[M – H]^−^	203.4	112.9/142.0	22/22	25/19	34/26	4.65
TEN	[M – H]^−^	413.4	141.1/271.3	14/14	23/20	12/16	11.78
ZEN	[M – H]^−^	413.4	141.1/271.3	14/14	23/20	12/16	12.37

### Calibration and quantification

SIDA was used for all toxins where ILS were available, namely DON, DON-3-G, 3-AcDON, T-2, HT-2, and ENN A1 of the *Fusarium* toxins, AOH, AME, and TeA of the *Alternaria* toxins, and AFB1, AFB2, AFG1, AFG2, OTA, and STC of the *Aspergill*us toxins. ENN A, ENN B, ENN B1, and BEA were quantified with response curves using [^15^N_3_]-ENN A1 as a structurally similar internal standard (IS). The other toxins, namely Fus X, NIV, and ZEN of the *Fusarium* toxins and TEN, ATX I, ALTP, AOH-3-G, AOH-3-S, and AME-3-S of the *Alternaria* toxins, were quantified via MMC.

Response curves were prepared for those toxins for which internal standards were available. The curves were created by mixing analytes (A) with their respective standards (S) (either ILS or IS) in specific amounts to obtain molar ratios n(A)/n(S) ranging from 0.01 to 100 (1:100, 1:50, 1:25, 1:10, 1:5, 1:2, 1:1, 2:1, 5:1, 10:1, 25:1, 50:1, 100:1). The absolute amount of ILS or IS was kept constant. For TeA, AOH, AME, DON, 3-AcDON, DON-3-G, T-2, HT-2, ENN A1, AFB1, AFB2, AFG1, AFG2, STC and OTA, their respective isotopologues [^13^C_6_,^15^N]-TeA, [^2^H_4_]-AOH, [^2^H_4_]-AME, [^13^C_15_]-DON, [^13^C_17_]-3-AcDON, [^13^C_21_]-DON-3-G, [^13^C_4_]-T-2, [^13^C_22_]-HT-2, [^15^N_3_]-ENN A1, [^13^C_17_]-AFB1, [^13^C_17_]-AFB2, [^13^C_17_]-AFG1, [^13^C_17_]-AFG2, [^13^C_18_]-STC, [^13^C_20_]-OTA were used as ILS. For ENN A, ENN B, ENN B1, and BEA, [^15^N_3_]-ENN A1 was used as IS for the whole group of depsipeptides. Following the LC–MS/MS measurement, the peak area ratios [A(A)/A(S)] were plotted against the corresponding molar ratios [n(A)/n(S)].

For toxins without an internal standard, MMC curves were obtained for the respective compounds by spiking the blank matrix with eight to 10 concentrations. The LOQ was used as the lowest spiking level, while the highest spiking level was at least 10 times higher.

The modified *Alternaria* toxins AOH-9-G, AOH-9-S, and AME-3-G were only included qualitatively in the method because the available amounts of these toxins were insufficient to generate an MMC for quantitation. However, while screening the 100 analyzed samples, we found none of those three modified *Alternaria* toxins.

### Method validation

As no blank sample devoid of all analytes was available, we selected a rice drink for the validation of ENN A, ENN A1, ENN B, and ENN B1, as well as BEA, and cashew-based products for all other toxins, both having the least toxin content from our sample set.

### LODs and LOQs

Limits of detection (LODs) and limits of quantification (LOQs) were determined according to the literature (24). The blank matrix was spiked in triplicate with the analytes and respective internal standards at four different concentration levels, starting at the estimated LOD value and increasing up to about 10 times the estimated LOD. Details about spiking concentrations can be found in Supplementary Table 2. All samples were worked up according to the optimized method. From the results of the measurements, the respective LOD was calculated using the intercept of the upper 95% confidence interval of the resulting matrix-matched calibration curve with the y-axis for addressing the critical signal value representing the LOD. For the respective LOQ, the following criteria were addressed and calculated as concentration levels: (i) significant distance of LOQ from LOD (ii) completeness of the recovery (80–120%), and (iii) quality of precision. The worst of these values was taken for the LOQ of the method.

### Recovery

To evaluate the recovery, the blank matrix was spiked in triplicate in five concentrations that reflected realistic toxin concentrations expected to be in the real samples. Starting from a value near the LOQ, increasing concentrations (up to 10 times the LOQ) were used. In Supplementary Table 2, the details of the spiking experiments are provided. After the sample work-up and LC–MS/MS analysis, the recovery was calculated as the ratio of the quantified amount of toxin divided by the spiked concentration, multiplied by 100.

### Precision

The blank matrix was spiked in triplicate with all analytes and corresponding standards and processed using the developed QuEChERS approach. For intra-day precision (*n* = 3), three samples were worked up on the same day, and for inter-day precision (*n* = 9), three samples were worked up on three different days. Measurements were performed in triplicate in consecutive weeks. Inter-injection precision (*n* = 10) was determined as the standard deviation of 10 successive injections of a toxin mixture containing all analytes and internal standards. The exact concentrations used are provided in Supplementary Table 2.

### Analysis of commercial products

A wide variety of 100 samples were bought in supermarkets in the region of Munich (Bavaria, Germany) or from online platforms for analysis. The sample set consisted of a variety of PBMAs, namely 31 oat-based, 24 based on other cereals (rice, spelt, millet, buckwheat), 31 nut-based (almond, cashew, hazelnut, walnut, tiger nut), and 14 others based on soy, pea, and hemp. In Supplementary Table 3, a complete overview of the analyzed samples is given. To guarantee homogeneity, all samples were intensely shaken before taking out an aliquot of 50 mL that was stored at −18 °C until analysis.

### Data analysis

The software LabSolutions version 5.118 (Shimadzu, Kyoto, Japan) was used to integrate the peak areas. Analyte concentrations, response curves, and linearity were calculated using Microsoft Excel 2021 (Microsoft Co., Redmond, WA, United States).

### Risk evaluation

The consumption of PBMAs was assessed for toddlers and adults. In terms of milk consumption, toddlers appear to be most relevant due to group size and vulnerability (low body weight). Adults were assessed for comparison, although their milk consumption is significantly lower. For these groups, we assumed that the same amount of PBMA completely replaced the mean consumption of bovine milk. For the bovine milk consumption, we took the data from the German national consumption survey II (NVS II) (25) for adults and the KIESEL study (26, 27) for toddlers (age 1–3 years). In detail, these consumption values were 1.92 g/kg bw per day and 12.5 g/kg bw per day, respectively.

When converting consumption and mycotoxin contents into exposure data, a critical aspect is the treatment of measurements that fall under the LOD or the LOQ. For our data set, we found a lot of values of this category, and thus, we faced a significant number of left-censored data. To address this, we used the substitution method as recommended by EFSA (28). For the lower bound (LB) scenario, all values below LOD and LOQ were replaced with 0. For the upper bound (UB) scenario, values below LOD were replaced with the LOD and values between LOD and LOQ were replaced with the LOQ. Following the substitution method, we obtained the mean LB and UB data for the mycotoxins in the respective types of PBMAs.

With these consumption values and the contamination mean data, we calculated the estimated daily intake (EDI) as: EDI [μg/(kg bw × day) = content (μg/kg) × consumption data per bw (kg/kg bw)].

As health-based guidance values (HBGV), we used the tolerable daily intake (TDI) of 1 μg/kg bw per day for the DON (group TDI) (29), the TDI of 0.02 μg/kg bw per day for the sum of T-2 and HT-2 (30), and the TDI of 1 μg/kg bw per day for ZEN (31).

Due to the lack of TDIs for the *Alternaria* toxins, we used the threshold for toxicological concern (TTC) of 2.5 μg/kg bw per day for TeA and TEN and 1.5 ng/kg bw per day for AOH and AME (32). For the latter two toxins, we calculated the sum of AOH, AOH-3-G, and AOH-3-S, and of AME and AME-3-S, respectively.

For the ENNs and BEA, for which no toxicological data are available, the sum of all compounds made up their EDI and was related to the TTC of TEN (2.5 μg/kg bw per day) due to structural analogy.

For AFB1, the margin of exposure (MoE) was calculated with respect to the benchmark dose lower confidence limit 10% (BMDL_10_) value of 0.4 μg/kg bw per day as follows: MoE = BMDL_10_/EDI.

## Results and discussion

A QuEChERS method for simultaneously analyzing different *Alternaria, Aspergillus, and Fusarium* toxins was successfully developed for 31 toxins and validated for 28 of them. The developed method was based on our previously published method for the analysis of 24 different *Alternaria* and *Fusarium* toxins in cereals and cereal-based products (14). While expanding the method, the *Alternaria* toxin ALTP and the *Aspergillus* toxins OTA, STC, AFB1, AFB2, AFG1, and AFG2 were added to the method. The modified *Alternaria* toxins AME-3-G, AOH-9-G, and AOH-9-S were not validated, as those toxins did not occur during the screening process of all 100 samples and, therefore, validation of those compounds was not considered a priority.

### Method development

During method development, we adjusted our existing work-up procedure to fit for liquid samples and the extraction of the newly added mycotoxins ALTP, OTA, STC, AFB1, AFB2, AFG1, and AFG2.

### Extraction

The nature of liquid samples enabled us to increase sample volume for better sensitivity. We tested the usage of 5 and 10 mL, and as 10 mL did not show a strong increase in matrix effects, we chose the higher volume, as it was used in the original QuEChERS method (23). Both 5 and 10 mL sample volumes are used frequently in the literature (5, 33–36).

The extraction was tested in sample/ACN volumetric ratios of 2:1, 1:1, and 1:2, as is frequently used in the literature (5, 34, 35, 37, 38). Of these ratios, 1:1 showed the best results in terms of signal intensity and allowed easy handling. To ensure enough extract volume for the subsequent clean-up steps, we used 11 mL of ACN instead of 10 mL.

We also tested the effects of FA on the new matrix and added 0, 0.1, 0.5, 1, and 2% FA for the extraction. In accordance with our previous outcomes, the addition of 1% FA showed the best results, allowing the protonation of TeA and thus leading to good extraction (39), while increasing the coextracted matrix only in low amounts. Besides being necessary for the extraction of TeA, the addition of small amounts of FA may also have additional benefits, like reducing the interaction of mycotoxins with matrix components like proteins (14, 40).

We also varied the extraction time at 350 min^−1^ (0, 10, 20, 30, 60, and 120 min), while for all extraction times, the samples were shaken intensely by hand for 30 s before placing them on the horizontal shaker. In agreement with the literature, all extraction times led to comparable results. To reduce the influence of the manual shaking, we decided to shake the samples for 10 min on the horizontal shaker. The short extraction time is well in line with other methods (5, 38), and often the extraction time is even further reduced (34, 35).

### QuEChERS clean-up

Based on our previous results indicating no need for other salt combinations, we used 1 g NaCl and 4 g MgSO_4_ as QuEChERS salts for all work-ups, as in the original published QuEChERS method (23). Opposing our previous results, adding 1% FA for the QuEChERS step did not prove beneficial for the analysis of TeA, and therefore was not included in the work-up.

### Defatting

After QuEChERS clean-up, we also tested defatting steps using cyclohexane. However, the defatting step was unnecessary as the combination of QuEChERS and d-SPE proved to be sufficient clean-up, and the defatting step coextracted some mycotoxins while not significantly reducing the matrix burden. Leaving out the defatting step is well in agreement with most literature known methods, except for one, where heptane is used for defatting (5).

### d-SPE clean-up

To further clean up the extract, we decided to test two d-SPE variants, PSA and MgSO_4,_ and the combination of PSA, C18, and MgSO_4_, as those two combinations showed the best results in our previous experiments. The results aligned with our previous studies, which showed the suitability of both d-SPE tubes for all toxins. However, as we included more mycotoxins in this method, which benefited from adding C18, we changed the d-SPE clean-up to the combination of PSA, C18, and MgSO_4_, allowing the best performance on average. Extraction times of 0, 5, 10, 15, 30, and 60 min were tested. While the clean-up was already good after 30 s of manual shaking (0 min), we chose 15 min as the extraction time, as it is the same time we use in our other method for solid samples. Therefore, it fits the workflow, and the longer extraction time should reduce errors. Accordingly, every extraction time starting at 5 min should be sufficient, with longer times than 15 min leading to no disadvantages, but being unnecessary. While some other methods do not employ further clean-up, as some mycotoxins may be adsorbed at the d-SPE materials (38), in our case, using d-SPE generally proved to be beneficial.

### Reconstitution solvent

A low volume of 200 μL revealed the best intensity and signal-to-noise ratio for all analytes, while matrix contamination was still acceptable. The low reconstitution volume is possible with the good clean-up achieved by QuEChERS and d-SPE. Ratios of 6:4 (v/v) for MeOH/water gave the best results for the re-solvation of analytes, matrix reduction, and peak shape, supporting our previously chosen solvent also for the six recently added mycotoxins (14). To prevent matrix precipitation during storage or at the autosampler temperature of 4 °C, the reconstituted extract was frozen for 30 min. Afterward, the sample was filtered with a PVDF filter before LC–MS/MS analysis. Again, our solvent ratio used for reconstitution is in line with the literature (38).

### Sample concentration of the final method

We achieved a simple, fast, and sensitive work-up approach, made possible by a high sample volume of 10 mL. We add 11 mL ACN to the sample, of which we take 9.5 mL after the QuEChERS clean-up to further cleanup via d-SPE. We finally put 8 mL to dryness and reconstitute it in a final volume of 200 μL. When assuming all toxins to be transferred into the ACN phase, we have an equivalent of 72.7% of the sample present in the final volume, which equals a concentration of the sample by a factor of 36.3. While other methods further dilute the sample (35, 41), some other methods achieve a similar concentration factor (37, 38), but most concentration factors in the literature are below ours.

### Calibration and quantitation

Linearity of the response functions of the analytes AOH, AME, TeA, AFB1, AFB2, AFG1, AFG2, OTA, STC, DON, DON-3-G, 3-AcDON, T-2, HT-2, ENN A, ENN A1, ENN B, ENN B1, and BEA in relation to their internal standards was verified with Mandel’s fitting test (42). The linear range encompassed molar ratios from 0.01 to 100 for most toxins, except for ENN A1 (0.01–20), HT-2 (0.02–100), AFB2 (0.01–50), and AFG2 (0.02–10). However, this was no problem for those toxins, as the analyte to IS ratio in all samples did not exceed the linear ratio.

The MMC curves for the analytes without internal standards were also checked for linearity using Mandel’s fitting test (42), resulting in the following calibration ranges: TEN (0.06–5 μg/L), ATX I (0.3–20 μg/L), ALTP (0.9–20 μg/L), AOH-3-S (0.06–20 μg/L), AME-3-S (0.015–20 μg/L), AOH-3-G (0.07–20 μg/L), NIV (6–200 μg/L), Fus X (1.2–50 μg/L) and ZEN (0.06–50 μg/L).

### Method validation

Our goal was to get a rough overview of the broad variety of PBMAs, and, therefore, we intended a general validation for all matrices. While a special validation for all matrices surely grants a higher accuracy, we are confident that our results represent a correct quantitative value. Ideally, a general validation for all types of examined drinks requires a matrix consisting of a similar major component. However, the major component of the drinks may strongly vary, and various combinations for a unified matrix are possible. Moreover, it was difficult to find a matrix free from all analytes, which led us to select rice and cashew-based products, as those showed the least toxin contamination. When comparing the intensity of internal standards in rice and cashew-based drinks, for most toxins, the matrix did not have a big impact on the analyte signal, which proved the performance of the chosen extraction method. Exceptions were ENN A, ENN A1, ENN B, and ENN B1, as well as BEA, which interestingly showed strong signal suppression while being measured in cashew-based products.

### LODs and LOQs

LODs and LOQs were determined according to the literature (24). Therefore, an analyte-free blank matrix was spiked in four concentrations. Finding a completely analyte-free matrix was challenging, so we validated the method in two different matrices. The LODs and LOQs of the ENNs and BEA were determined in a rice-based drink, because the cashew drink used to validate all other toxins was not entirely free of these toxins. However, even the rice-based drink contained traces of BEA in concentrations near the LOD, and thus, the native BEA concentration was quantified via standard addition, and the calculated value was used as the lowest concentration level for the determination of the LODs and LOQs. As the calculated LOD is well in line with the other ENNs and in agreement with our previous results (14), this unconventional approach appeared reasonable.

The results of the LOD and LOQ determination are summarized in Table 2. The LODs ranged from 0.0009 μg/L (ENN A1) to 1.15 μg/L (NIV), while the LOQs ranged from 0.0032 μg/L (ENN A1) to 3.74 μg/L (NIV). The AFs, AME, and ENNs showed remarkably low LODs and LOQs, which may be caused by a good ionization efficiency without many matrix interferences. In the case of the ENNs, the formed ammonium adducts are especially sensitively detected in the MS.

**Table 2:** Limits of detection (LODs), limits of quantification (LOQs), relative standard deviation (RSD) values, and recoveries for all toxins in the blank matrix.

Analyte	LOD (μg/L)	LOQ (μg/L)	Precision (RSD) (%)			Recovery (%)				
			Inter-injection (*n* = 10)	Intra-day (*n* = 3)	Inter-day (*n* = 9)	Level 1	Level 2	Level 3	Level 4	Level 5
AOH	0.026	0.094	3.6	0.8	2.2	101.6	104.1	103.7	103.9	95.9
AOH-3-G	0.032	0.120	1.2	0.2	1.4	90.5	97.6	98.1	101.1	100.0
AOH-3-S	0.008	0.024	1.1	0.6	2.8	103.1	82.7	109.1	100.2	100.0
AME	0.002	0.006	1.8	2.0	2.6	100.5	97.0	98.6	101.0	100.3
AME-3-S	0.004	0.014	8.6	1.1	2.7	108.3	108.0	97.3	102.9	99.9
TeA	0.027	0.098	2.5	1.0	1.6	101.6	101.8	100.8	101.2	101.4
ATX I	0.046	0.160	1.2	0.8	3.7	104.6	103.2	100.3	98.3	100.4
ALTP	0.130	0.520	3.7	2.0	2.6	99.5	99.7	102.1	100.2	99.9
TEN	0.013	0.048	1.0	1.9	4.0	106.5	100.5	90.0	101.7	99.9
DON	0.17	0.64	2.8	2.1	2.8	100.6	103.6	103.6	102.2	101.9
DON-3-G	1.07	3.72	3.3	1.2	2.7	97.6	97.2	100.8	101.4	99.7
3-AcDON	0.10	0.37	4.5	0.2	2.1	100.6	96.4	104.6	96.7	103.9
NIV	1.15	3.74	3.4	1.0	2.4	93.7	95.8	95.2	104.7	99.8
HT-2	0.05	0.17	4.2	1.4	2.9	100.2	102.6	102.0	96.8	99.8
FusX	0.20	0.69	3.2	3.2	4.4	94.4	101.7	103.7	98.7	100.3
T-2	0.01	0.04	5.2	0.3	1.5	103.9	104.0	97.9	95.3	96.6
ZEN	0.02	0.05	3.2	1.9	3.7	104.6	94.5	104.9	101.6	100.1
ENN A	0.0016	0.0060	3.1	7.5	7.8	86.1	83.4	81.8	99.9	91.0
ENN A1	0.0009	0.0032	2.5	0.8	1.1	104.2	103.9	99.8	100.9	101.2
ENN B	0.0010	0.0036	2.6	1.3	11.3	99.3	95.8	100.3	97.9	99.4
ENN B1	0.0010	0.0032	2.6	0.8	2.5	109.2	109.4	103.2	100.4	101.5
BEA	0.0017	0.0052	2.8	2.5	4.7	104.5	94.5	111.0	101.8	84.8
OTA	0.050	0.240	1.2	1.2	3.1	99.8	102.0	99.8	100.5	99.7
STC	0.0012	0.0044	1.6	1.7	2.1	102.6	100.4	104.6	102.2	97.9
AFB1	0.0038	0.013	4.3	1.6	2.1	99.6	104.7	101.2	103.3	101.0
AFB2	0.0039	0.014	3.5	1.9	2.6	97.00	103.5	102.4	103.6	98.4
AFG1	0.0062	0.025	3.7	2.1	2.2	100.3	104.4	100.6	99.7	102.5
AFG2	0.037	0.14	4.7	1.4	3.7	101.5	98.7	99.5	103.8	103.9

Recovery values of each spiking level were determined as the mean value of three replicates in triple injection.

In recent years, a few multi-mycotoxin methods focusing on plant-based beverages have been developed (5, 33, 35, 36, 38). In general, we achieved similar or better sensitivity for most analytes. Our distinguishing feature was, on the one hand, the inclusion of modified mycotoxins (e.g., DON-3-G) and thereof, especially modified *Alternaria* toxins, for quantitative (AOH-3-G, AOH-3-S, and AME-3-S) and qualitative (AOH-9-G, AOH-9-S, and AME-3-G) analyses and, on the other hand, the inclusion of TeA, the most frequently occurring *Alternaria* toxin. Additionally, we were able to analyze the two perylenquinones ATX I and ALTP using our method. Those two *Alternaria* toxins are not usually part of multi-mycotoxin methods and have not yet been analyzed in other studies focusing on PBMAs. Setting a priority on TeA provides the first opportunity to get occurrence data of the most frequently occurring *Alternaria* toxin. The sensitivity for the other *Alternaria* toxins was good and similar to those reported in the literature.

The sensitivity for *Fusarium* toxins that form ammonium adducts like T-2, HT-2, ENN A, ENN A1, ENN B1, ENN B, and BEA was similar or better than in other methods. Moreover, the LOQs for DON, NIV, 3-AcDON, Fus X, and ZEN were low and in a similar range compared to methods in the literature. The LOQ of DON-3-G was slightly higher but still sufficiently low. The decreased sensitivity may be caused by in-source fragmentation under the measurement conditions used, as other, comparable methods are more sensitive using different instrumentation (5, 33, 35, 36, 38).

The *Aspergillus* toxins AFB1, AFB2, AFG1, AFG2, STC, and OTA have sufficiently low LOQs between 0.013 and 0.14 μg/L, with AFG2 showing 10 times higher LOQs, probably caused by matrix interferences. Our sensitivity is well in line with other literature methods (5, 33, 35, 36) and far below the MLs for AFs and OTA (8).

Overall, the method proved to be a sensitive quantification approach for all analytes, being sensitive enough to check compliance with MLs and allow a comprehensive risk assessment.

### Recovery

The recovery was determined by spiking each analyte in triplicate at three to four concentrations in the blank matrix. The lowest concentration was set at the LOQ, while the highest concentration was chosen to cover a reasonably high range, reflecting the expected levels in the samples. As shown in Table 2, recoveries were between 82.7 and 111.0% for all analytes, meeting the acceptance criteria of 70–120% (24).

### Precision

Intra-day, inter-day, and inter-injection precision were determined by calculating the RSD of every analyte after a defined number of repeated measurements. Intra-day precision was evaluated by preparing one sample in triplicate on the same day, and the range was from 0.2 to 7.5%. Inter-day precision was generated by analyzing one sample in triplicate weekly for 3 weeks and ranged from 1.1 to 11.3%. Inter-injection precision was calculated after 10 times injecting a toxin mixture in solvent (MeOH/H_2_O, v/v) containing all analytes. Inter-injection RSD was between 0.5 and 8.6%, thus showing the system’s stability for most analytes. All obtained precisions are shown in Table 2.

### Quantitation of mycotoxins in PBMAs

We analyzed a total of 100 samples of PBMAs, mainly from local retailers, but also from online platforms (for a complete overview, please refer to Supplementary Table 3). The raw materials of the PBMAs consisted of a huge variety of plant materials, and each drink was mainly made from one source. However, some were also based on the mixture of plant materials or included minerals or other supplements. According to our sample set, we classified our samples into PBMAs based on (a) oats, (b) other cereals, (c) nuts, and (d) other plant materials. As expected, some of the mycotoxins were rather specific to the plant origin of the PBMA, which will be outlined below when dealing with the single PBMA classes. Irrespective of these classes, the following general results were found for all the groups:

The mean contents detailed below refer to the mean of all samples above the LOQ. In contrast to this, for the exposure assessment, the LB and UB approach was applied as detailed in the methods section.

Among the *Fusarium* depsipeptides, we found a very frequent contamination with BEA being detectable in 83% of all samples, followed by ENN B (81%), ENN A1 and B1 (both 80%), and ENN A (27%). Of these, the content was highest for ENN B with 1.4 μg/L in mean and a maximum content of 18.4 μg/L in a hazelnut drink. These results were somewhat contradictory to the literature (41), which reported values for oat drinks ranging between 5.5 and 26 μg/L ENN B and soy drinks ranging between 11 and 22 μg/L ENN B. Concerning the mean contents, the distribution in our samples was in descending order: ENN B, ENN B1, ENN A1, BEA, and ENN A.

ZEN, another *Fusarium* toxin, was not detectable in most of the samples, and in only 5 samples it was quantifiable.

In the group of *Alternaria* toxins, TeA was detected in almost all samples (93%) with an overall mean content of 6 μg/L, and the maximum being 86 μg/L in a hazelnut drink. TEN was also frequently detected (79%) with a low overall mean of 0.11 μg/L.

OTA was only detectable in 12 samples, and none were quantifiable (LOQ of 0.15 μg/L). This result is well in line with the recent report on mycotoxins in drinks based on soy, almonds, and oats, where OTA was neither detectable in any drink (5).

### Oat drinks

For the first class of PBMAs, namely the oat drinks, 31 products were analyzed. According to the labeling, the oat content ranged from 7.8 to 15% oats. Some products were also labeled as fermented; some included ingredients like cocoa or curcuma; and some were made from whole oats or whole oat flour. Frequent further ingredients were calcium, vitamin B2, vitamin D, and vitamin B12. Of all samples containing oats, eight were labeled as having been manufactured from organic oats. The results for the oat drinks are detailed in Table 3. Among the *Fusarium* toxins, we found a frequent contamination (87%) with type B trichothecenes from the DON group, with a low mean sum of DON, DON-3-G, and 3-AcDON of around 1.5 μg/L. This was expected due to the general low occurrence of DON in oats, although we also found one sample with a high sum content of around 30 μg/L. The distribution between DON and its modifications was very variable, with DON being the most prevalent component. However, in mean, DON-3-G accounted for 40% of the sum content.

**Table 3:** Mycotoxin contents in 31 plant-based milk alternatives based on oats.

Analyte	Positive samples (number/percent of all samples)	Mean of all positive samples (μg/L)	Maximum content (μg/L)
DON	27/87%	1.52	19.4
DON-3-G	5/16%	0.982	11.6
3-AcDON	5/16%	0.024	0.371
T-2	31/100%	0.299	1.67
HT-2	30/97%	0.833	7.94
ZEN	5/16%	0.015	0.399
ENN A1	31/100%	0.168	1.43
ENN B1	31/100%	0.761	5.78
ENN A	11/35%	0.128	1.27
ENN B	30/97%	3.25	17.4
BEA	31/100%	0.177	1.12
TeA	30/97%	1.12	7.21
AOH	0/0%	0	0
AOH-3-G	0/0%	0	0
AOH-3-S	0/0%	0	0
AME	7/23%	0.001	0.006
AME-3-S	0/0%	0	0
TEN	28/90%	0.079	0.257
OTA	2/6%	0.003	0.050
STC	9/29%	0.005	0.078
AFB1	1/3%	0.002	0.059
AFB2	0/0%	0	0
AFG1	1/3%	0.0002	0.006

Regarding the type A trichothecenes, the oat drinks were the most frequently contaminated products, with HT-2 being three times as high as T-2 in almost all samples. In mean, the samples above the LOQ contained 0.30 and 0.83 μg/L of T-2 and HT-2, and in maximum 1.7 and 7.9 μg/L, respectively. The high contamination frequency is well in line with the recent report on mycotoxins in drinks based on oats (5). However, the sum content of 2.1 μg/L in maximum and 0.4 μg/L in the mean stated therein was significantly lower than in our samples.

Concerning the other toxins, a contamination was generally either absent or low. Furthermore, the organic oat drinks did not show significantly different toxin content than the conventional oat drinks.

### Cereal drinks

The second class of PBMAs, namely those made from cereals other than oats, often also in mixture with different materials, drinks from rice, spelt, millet, and the pseudocereal buckwheat were analyzed (Table 4).

**Table 4:** Mycotoxin contents in 24 plant-based milk alternatives based on cereals other than oats.

Analyte	Positive samples (number/percent of all samples)	Mean of all positive samples (μg/L)	Maximum content (μg/L)
DON	10/32%	0.347	2.97
T-2	12/39%	0.018	0.117
HT-2	8/26%	0.055	0.472
ZEN	8/26%	0.008	0.065
ENN A1	14/45%	0.015	0.117
ENN B1	14/45%	0.085	0.707
ENN A	3/10%	0.001	0.011
ENN B	16/52%	0.433	3.47
BEA	23/74%	0.010	0.069
TeA	24/77%	9.01	62.0
AOH	5/16%	0.184	2.22
AOH-3-G	3/10%	0.056	0.611
AME	19/61%	0.012	0.048
AME-3-S	3/10%	0.002	0.021
TEN	22/71%	0.075	0.216
OTA	1/3%	0.002	0.050
STC	15/48%	0.026	0.417
AFB1	7/23%	0.012	0.104
AFB2	5/16%	0.001	0.004
AFG1	1/3%	0.0003	0.006

Contamination with trichothecenes mainly occurred in spelt drinks, whereas the contents were generally lower than those in oat drinks. In this product class, TeA contents were highest in millet-based drinks, which we had already expected from our previous reports on millet-based infant foods (43). For buckwheat drinks, we found it interesting that in all samples, AOH-3-G exceeded the content of AOH, which has not been reported before. Rice drinks, which comprised most of this class’s products, revealed a rather low contamination of all mycotoxins under study.

### Nut drinks

The third product class consisted of nuts, including tiger nuts and chestnuts, and the summary of the results is shown in Table 5. Trichothecene contamination was rather low, but we found significant contents of *Alternaria* toxins and AFs in this class. Among the former, the contents of TeA and AOH were outstanding in hazelnut drinks. Although their frequent contamination with mycotoxins has already been reported (15), the extent of contamination with AOH was still surprising. The presence of AFs in this product class was expected, and the drinks made from almonds were frequently contaminated with this group of mycotoxins. This result is well in line with the recent publication on the safety of drinks made from almonds, which reported in almost all samples the detection of AFB1 (5). Another surprisingly high frequency and abundance of AF contamination was found in drinks from tiger nuts. This product type has been popular in Spain for centuries and is generally called “Horchata de Chufa” (3). All four investigated products originated from Spain and contained AFB1, AFB2, and AFG1, with the highest mean content of AFB1 (0.67 μg/L) for all AFs and in all types of samples. This was particularly striking as an earlier report from 2006 detected AFB1 in only one of 22 tiger-nut-based drinks (44). AFG2 was not detectable in any of the samples, possibly due to its lower abundance than the other AFs (15) and our method’s high LOD for this compound (0.06 μg/L).

**Table 5:** Mycotoxin contents in 31 plant-based milk alternatives based on nuts.

Analyte	Positive samples (number/percent of all samples)	Mean of all positive samples (μg/L)	Maximum content (μg/L)
DON	1/3%	0.131	4.07
T-2	3/10%	0.005	0.074
HT-2	1/3%	0.006	0.189
ZEN	13/42%	0.008	0.055
ENN A1	21/68%	0.281	5.70
ENN B1	21/68%	0.681	13.8
ENN A	7/23%	0.028	0.750
ENN B	23/74%	0.916	18.4
BEA	17/55%	0.007	0.102
TeA	26/84%	9.96	85.9
AOH	10/32%	0.351	4.40
AOH-3-G	2/6%	0.002	0.032
AOH-3-S	5/16%	0.006	0.085
AME	25/81%	0.033	0.267
AME-3-S	11/35%	0.007	0.098
TEN	19/61%	0.038	0.155
OTA	6/19%	0.010	0.050
STC	16/52%	0.014	0.284
AFB1	16/52%	0.069	0.674
AFB2	7/23%	0.007	0.063
AFG1	8/26%	0.016	0.184

### Other drinks

The fourth and last class of PBMAs was based on other plant materials, such as hemp and legumes like peas and soybeans. Interestingly, the samples showed a very low frequency of contamination, with the highest number of detections for the ENNs and TeA, albeit at very low mean and maximum concentrations (Table 6). Notably, two of the three PBMAs made from hemp seeds showed quantifiable contents of ZEN, albeit with a low maximum content of 0.41 μg/L. In contrast to the literature (45), we observed no unusually high levels of *Alternaria* toxins.

**Table 6:** Mycotoxin contents in 14 plant-based milk alternatives based on soy beans, peas, and hemp.

Analyte	Positive samples (number/percent of all samples)	Mean of all positive samples (μg/L)	Maximum content (μg/L)
DON	2/14%	0.031	0.170
T-2	2/14%	0.012	0.121
ZEN	3/21%	0.005	0.017
ENN A1	11/79%	0.004	0.012
ENN B1	11/79%	0.017	0.053
ENN A	6/43%	0.101	1.08
ENN B	10/71%	0.055	0.171
BEA	9/64%	0.005	0.020
TeA	10/71%	0.208	0.699
AOH	0/0%	0	0
AOH-3-G	0/0%	0	0
AOH-3-S	0/0%	0	0
AME	2/14%	0.001	0.009
AME-3-S	0/0%	0	0
TEN	7/50%	0.021	0.098
OTA	2/14%	0.009	0.050
STC	1/7%	0.001	0.012
AFB1	0/0%	0	0
AFB2	0/0%	0	0
AFG1	0/0%	0	0

### Risk assessment of PBMAs

#### General considerations

To assess the risk for the consumers of PBMAs, we had to make several assumptions and consider some limitations. First, several mycotoxins were not detectable or quantifiable in many drinks, meaning the data for many drinks are left-censored. Therefore, we compared the UB mean with the LB mean for all types of drinks, and we considered the data reliable only if the mean UB exposure did not differ significantly from the respective LB value. If this was not the case, we only considered, if ever, the maximum levels for a worst-case scenario. Secondly, the consumption data for PBMA is scarce when calculating exposure. Therefore, in a conservative approach, we assumed that the same amount of the respective PBMA completely replaced the mean consumption of bovine milk.

As the reference group for risk assessment, we chose German adults. For the most vulnerable age group, toddlers (1–3 years) were chosen, as their daily milk consumption per body weight is reported to be highest for all population groups (12.5 g/kg bw per day) (26, 27). By contrast, the daily milk consumption per body weight of adults is around a factor of 6 lower (2 g/kg bw per day) (25). Infants (<1 year) were not further considered, as replacing breast milk or infant formula with PBMA is not recommended.

For the risk evaluation of single types of PBMA, we grouped the samples from the same raw materials and assessed these groups separately. We chose this approach as the consumers most likely do not mix the different drinks due to their significantly different sensory attributes, and, therefore, the single consumers may mostly prefer one type over the others. For the single mycotoxins and toxin groups, we applied the following considerations:

DON was assessed as the sum with its modifications, DON-3-G and 3-AcDON, and related to the group TDI (29). The contents of T-2 and HT-2 were always assessed as their sum for comparison with their sum TDI (30). All ENNs and BEA were also evaluated as the sum of all single compounds. Until now, a TDI has not been elaborated for these compounds as only NOAEL data are available for poultry, and the EFSA Contam Panel found the data insufficient for further assessment (46). However, following the assumptions made for the *Alternaria* toxins and given the structural similarity with TEN, one may classify the ENNs and BEA similarly and assign them a TTC of 2.5 μg/kg bw per day.

The benzopyrones AOH and AME were always evaluated as their respective sum, including their modifications AOH-3-G, AOH-3-S, and AME-3-S. This aligns with the general assumption that the conjugates can completely release the non-conjugated compounds during human digestion. As health-based guidance values, the TTC of AOH and AME was chosen (32).

For the *Alternaria* toxin TeA, we found no sample to exceed the indicative level for cereal-based infant food of 500 μg/kg (12). However, when considering the dilution with water in the drink, some raw materials most likely exceeded this indicative level, as can be expected for two hazelnut drinks and one hemp drink. However, the EDI would amount to only 67% of the TTC of TeA for toddlers, even for the hazelnut drink with the maximum TeA content of 85 μg/L.

For TEN, the same TTC as for TeA was applied, but the EDI, even in the worst-case scenario with the maximum contaminated sample, is less than 1% of the TTC for toddlers.

Among the AFs, only AFB1 was evaluated as it is the most carcinogenic compound and the only one for which a BMDL_10_ exists.

OTA was not evaluated as its LOQ was not exceeded in any of the samples, although it was detectable in 12 samples. For applying the MoE concept, the method would need to be more sensitive, but this was a compromise to detect the other mycotoxins more efficiently.

For exposure assessment of the different types of PBMAs, the mean LB values were generally considered. In some cases, the UB mean values or MLs were mentioned.

All evaluations were based on the respective HBGV (TDI, TTC) or point of departure (BMDL_10_) and, if available, on MLs for the respective raw materials from which the PBMAs have been produced.

#### Risk assessment of oat drinks

For the risk assessment of oat drinks, the overall occurrence of T-2 and HT-2 was expected, but it also appeared alarming in the detailed view. When considering the high consumption per bodyweight for toddlers, the EDI reached almost 70% of the TDI (0.02 μg/kg bw) for the mean of all samples, and the maximum containing sample exceeded the TDI by a factor of 5. In this regard, it appears critical that one of the oat products has been particularly advertised for children above 1 year. Although this product’s T-2 and HT-2 content was lower than the mean of all oat drinks, with its consumption, toddlers would reach 29% of the TDI, which is not advisable. A similar conclusion has been drawn in the recent publication on the safety of plant-based drinks (5). In that study, the authors based their assessment on adults with an average body weight of 65 kg. Assuming a high consumption of 500 mL, the drink with the highest T-2 and HT-2 content would account for 81% of the TDI. This consumption per bodyweight is similar to our mean consumption of toddlers per bodyweight. As the T-2 and HT-2 contents in our study were significantly higher, we assessed the exposure as even more critical.

A further risk assessment of PBMAs has recently been published by the German Federal Institute for Risk Assessment (BfR) (26), which was based on the previously published contamination data (5). The difference between the two latter risk assessments was the use of different consumption data. The BfR used recent data from the Children’s Nutrition Survey to Record Food Consumption (KiESEL) (27). With these data, the BfR calculated that 17% of the TDI was reached by the age group of 1–3 years for the mean contamination level and the mean consumption. For the product with the maximum contamination level, 93% of the TDI would be reached at the mean consumption level. However, as the BfR stated that their contamination data lacked representativeness and they could not consider additional T-2 and HT-2 exposure in the realistic scenario of oat flakes consumed together with oat drink, the BfR was not able to draw a conclusion on the long-term risk from oat-based drinks. Therefore, the BfR called for further occurrence data (26). With our report, we here present additional data, which are even higher than the latter and underline the possible risks conferred by the consumption of oat drinks by toddlers.

A different perspective would be the assessment according to the new MLs of T-2 and HT-2 in the EU (9). When considering the sum ML of 10 μg/kg for processed cereal-based foods for infants and young children, even the sample with the maximum contamination would meet this regulation. Moreover, as the ML for milling products of oat is 100 μg/kg, even the raw materials used for producing the oat drinks would be in line with the current regulations.

The trichothecenes of the DON group appeared less critical, with the highest contaminated sample reaching 39% of the group TDI for DON.

#### Risk assessment of cereal drinks and other drinks

In the product class of cereals other than oats, most products appeared unsuspicious. The drinks made from spelt contained low amounts of toxins from the DON group, and the intake of which would result in 1.6% of the group TDI for toddlers. Similarly, the T-2 and HT-2 content would yield only 10% of the respective TDI. For rice and soy drinks, the contents of mycotoxins did not result in any significant risk for the age groups under study. Noteworthy are the hemp drinks, the only type in the sample set, where ZEN was detectable in all samples. However, the contents did not appear critical, as even for the maximum content, just 2% of the TDI for toddlers would be reached.

#### Risk assessment of nut drinks

For the nut-based PBMAs, none of the *Fusarium* toxins can be seen as a risk. A risk evaluation is not easy in the case of the very frequently occurring ENNs, as an HBGV has not yet been established. However, one could apply a TTC value like that for the structurally similar TEN, and, thus, a maximum coverage of 19% of this value would be reached for toddlers drinking the hazelnut drink with the highest ENN contents.

If the whole set of nut-based drinks is considered, for the *Alternaria* toxin AOH, the EDI for toddlers LB is 180% of its TTC, which has to be seen as critical. Also, evaluating the AFB1 risk is critical, as the MoE for toddlers LB is as low as 464, thus far below the criterion of 10,000, and calling for measures to reduce the exposure. Even adults could be at risk with an MoE of 3,021.

However, the most popular nut-based PBMA is that made from almonds (47), which is of special importance in this regard. The MoE from the mean AFB1 content of the products (LB) was found to be as low as 1,940 for toddlers, i.e., in a range that calls for management action with high priority. The MoE exceeded 12,000 for adults, meaning a lower need for priority measures than for toddlers. The assessment for the age group of 1–3 years is in line with that from the BfR, which calculated an MoE of 2,539 for the mean contamination level and the mean consumption of almond drinks by this age group. Literally, “the BfR concludes that regular consumption of almond drinks with such AF levels may cause health impairments in children in the age group from 0.5 to <6 years with a medium likelihood” (26).

Concerning AFs, the tiger nut containing PBMAs were even more highly contaminated, and the MoE culminated at 69 for toddlers and 450 for adults. However, as these products are not commonly consumed and are more or less regional specialties, the health impact on the German population does not seem to be critical.

Another type of product that was relevant concerning *Alternaria* toxins was the hazelnut drinks. In all three analyzed products, AOH and AME were quantifiable, with the mean AOH and AME amounting to an EDI of almost 1,070 and 92% of the TTCs for toddlers, respectively. Although the values of the TTC have to be seen as a rough criterion, the exceedance of at least one magnitude is alarming.

## Conclusion

We successfully modified our existing multi-mycotoxin method (14) to be used for liquid samples. Six more mycotoxins relevant to PBMAs were included in the method. The method was thoroughly validated and displayed high sensitivity for all analytes.

Our results revealed a frequent contribution of the PBMAs to the general exposure to mycotoxins, and some of them have to be seen as critical for consumption by toddlers from a respective risk evaluation. In particular, the PBMAs from oats, almonds, hazelnuts, and tiger nuts were evaluated as a risk for this age group of consumers. This result is even more striking as all products would meet the current food regulations for mycotoxins. This points out that the existing legislation does not fit these new products that are becoming increasingly popular. Authorities are called to take action to protect the consumer against potential health issues originating from these products.

The other types of PBMAs were not that critical concerning mycotoxins, but that does not mean that these products can be recommended for toddlers in general, because there are further critical components to consider. For rice, the content of arsenic has to be considered. Given the reported and expected arsenic contents in rice drinks (48), the general recommendation of not using rice and products thereof for infants and children is supported (49). Soy drinks also did not show a risky mycotoxin contamination, but likewise, they are not recommended for infants and children due to their content of phytoestrogens (50). Interestingly, the lowest contamination with mycotoxins has been found for cashew drinks, but about consumer acceptance, these are the least favored ones compared to oat and almond drinks, being mostly preferred (47).

Our main conclusions from our current results are as follows: First, that we question prior publications [e.g., (51)], which highlighted the benefits of these drinks without considering mycotoxins and other contaminants adequately. Moreover, we think the BfR assessment of a “medium likelihood” that almond drinks cause health impairments in toddlers is an understatement.

Our second main conclusion is that the risk management authorities are called for action by urgently updating the respective mycotoxin regulation and initiating a rigorous control of mycotoxin contamination in PBMAs by the respective official laboratories.

## Data Availability

Data supporting this article have been included as part of the Supplementary material. Further information will be made available upon request from the corresponding author.

## References

[1] Markets and Markets (2023) Dairy alternatives market by source (soy, almond, coconut, oats, hemp), application (Milk, yogurt, ice creams, cheese, creamers), distribution channel (retail, online stores, foodservice), formulation and region - global forecast to 2028. Available online at: https://www.marketsandmarkets.com/Market-Reports/dairy-alternatives-market-677.html

[2] Khanpit V Viswanathan S Hinrichsen O. Environmental impact of animal milk vs plant-based milk: critical review. J Clean Prod. (2024) 449:141703. doi: 10.1016/j.jclepro.2024.141703

[3] Rios M Tinitana F Jarrín-V P Donoso N Romero-Benavides JC. “Horchata” drink in southern Ecuador: medicinal plants and people’s wellbeing. J Ethnobiol Ethnomedicine. (2017) 13:18. doi: 10.1186/s13002-017-0145-z, 28279218 PMC5345160

[4] Chalupa-Krebzdak S Long CJ Bohrer BM. Nutrient density and nutritional value of milk and plant-based milk alternatives. Int Dairy J. (2018) 87:84–92. doi: 10.1016/j.idairyj.2018.07.018

[5] Gützkow KL Lencioni A Schwake-Anduschus C Müller A Kabisch J Grundmann VL . Comprehensive investigation of undesired substances and microbial contamination in plant-based drinks. Food Control. (2024) 166:110599. doi: 10.1016/j.foodcont.2024.110599

[6] Alconada TM Moure MC Ortega LM. Fusarium infection in wheat, aggressiveness and changes in grain quality: a review. Vegetos. (2019) 32:441–9. doi: 10.1007/s42535-019-00054-z

[7] Kovalsky P Kos G Nährer K Schwab C Jenkins T Schatzmayr G . Co-occurrence of regulated, masked and emerging mycotoxins and secondary metabolites in finished feed and maize-an extensive survey. Toxins. (2016) 8. doi: 10.3390/toxins8120363, 27929415 PMC5198557

[8] European Commission (2023). Commission regulation (EU) 2023/915 of 25 April 2023 on maximum levels for certain contaminants in food and repealing regulation (EC) No 1881/2006 (Luxembourg City: Text with EEA relevance).

[9] European Commission (2024). Commission regulation (EU) 2024/1038 of 9 April 2024 amending regulation (EU) 2023/915 as regards maximum levels of T-2 and HT-2 toxins in food (Luxembourg City: text with EEA relevance).

[10] Gruber-Dorninger C Novak B Nagl V Berthiller F. Emerging mycotoxins: beyond traditionally determined food contaminants. J Agric Food Chem. (2017) 65:7052–70. doi: 10.1021/acs.jafc.6b03413, 27599910

[11] Vaclavikova M Malachova A Veprikova Z Dzuman Z Zachariasova M Hajslova J. ‘Emerging’ mycotoxins in cereals processing chains: changes of enniatins during beer and bread making. Food Chem. (2013) 136:750–7. doi: 10.1016/j.foodchem.2012.08.031, 23122123

[12] European Commission (2022). Commission recommendation (EU) 2022/553 of 5 April 2022 on monitoring the presence of Alternaria toxins in food, Luxembourg City.

[13] Rychlik M Humpf H-U Marko D Dänicke S Mally A Berthiller F . Proposal of a comprehensive definition of modified and other forms of mycotoxins including “masked” mycotoxins. Mycotoxin Res. (2014) 30:197–205. doi: 10.1007/s12550-014-0203-5, 24962446 PMC4202116

[14] Dick F Dietz A Asam S Rychlik M. Development of a high-throughput UHPLC-MS/MS method for the analysis of fusarium and Alternaria toxins in cereals and cereal-based food. Anal Bioanal Chem. (2024) 416:5619–37. doi: 10.1007/s00216-024-05486-4, 39222085 PMC11493838

[15] Varga E Glauner T Berthiller F Krska R Schuhmacher R Sulyok M. Development and validation of a (semi-)quantitative UHPLC-MS/MS method for the determination of 191 mycotoxins and other fungal metabolites in almonds, hazelnuts, peanuts and pistachios. Anal Bioanal Chem. (2013) 405:5087–104. doi: 10.1007/s00216-013-6831-3, 23471368 PMC3656230

[16] Schneidemann-Bostelmann S Otting Y Dick F Lefever T Asam S Rychlik M. Development and validation of an UHPLC-MS/MS multi-method for the analysis of mycotoxins in plant-based meat, cheese, and fish alternatives as the basis for risk assessment. Mycotoxin Res. (2024) 10.1007/s12550-026-00636-2 PMC12868108 41632390

[17] Scheibenzuber S Dick F Bretträger M Gastl M Asam S Rychlik M. Development of analytical methods to study the effect of malting on levels of free and modified forms of Alternaria mycotoxins in barley. Mycotoxin Res. (2022) 38:137–46. doi: 10.1007/s12550-022-00455-1, 35396694 PMC9038834

[18] Asam S Konitzer K Schieberle P Rychlik M. Stable isotope dilution assays of alternariol and alternariol monomethyl ether in beverages. J Agric Food Chem. (2009) 57:5152–60. doi: 10.1021/jf900450w, 19530709

[19] Asam S Liu Y Konitzer K Rychlik M. Development of a stable isotope dilution assay for tenuazonic acid. J Agric Food Chem. (2011) 59:2980–7. doi: 10.1021/jf104270e, 21370870

[20] Asam S Rychlik M. Synthesis of four Carbon-13-labeled type a Trichothecene mycotoxins and their application as internal standards in stable isotope dilution assays. J Agric Food Chem. (2006) 54:6535–46. doi: 10.1021/jf061347+, 16939307

[21] Hu L Rychlik M. Biosynthesis of 15N3-labeled enniatins and beauvericin and their application to stable isotope dilution assays. J Agric Food Chem. (2012) 60:7129–36. doi: 10.1021/jf3015602, 22734473

[22] Frank O Kreissl JK Daschner A Hofmann T. Accurate determination of reference materials and natural isolates by means of quantitative (1)h NMR spectroscopy. J Agric Food Chem. (2014) 62:2506–15. doi: 10.1021/jf405529b, 24559241

[23] Anastassiades M Lehotay SJ Stajnbaher D Schenck FJ. Fast and easy multiresidue method employing acetonitrile extraction/partitioning and “dispersive solid-phase extraction” for the determination of pesticide residues in produce. J AOAC Int. (2003) 86:412–31. doi: 10.1093/jaoac/86.2.412, 12723926

[24] Vogelgesang J Hädrich J. Limits of detection, identification and determination: a statistical approach for practitioners. Accred Qual Assur. (1998) 3:242–55. doi: 10.1007/s007690050234

[25] Heuer T Krems C Moon K Brombach C Hoffmann I. Food consumption of adults in Germany: results of the German National Nutrition Survey II based on diet history interviews. Br J Nutr. (2015) 113:1603–14. doi: 10.1017/S0007114515000744, 25866161 PMC4462161

[26] German Federal Institute for Risk Assessment (2024). Mycotoxins in plant-based drinks: more data required; results of a study by the max Rubner institute and their relevance for risk assessment; BfR-opinion no. 029/2024 of 15. June 2024. Bundesbehörden und Einrichtungen im Geschäftsbereich des Bundesministeriums für Ernährung und Landwirtschaft (Berlin: BMEL).

[27] Nowak N Diouf F Golsong N Höpfner T Lindtner O. KiESEL – the children’s nutrition survey to record food consumption for the youngest in Germany. BMC Nutr. (2022) 8:64. doi: 10.1186/s40795-022-00527-6, 35836299 PMC9284799

[28] EFSA. Management of left-censored data in dietary exposure assessment of chemical substances. EFS2. (2010) 8:1557. doi: 10.2903/j.efsa.2010.1557

[29] EFSA. Risks to human and animal health related to the presence of deoxynivalenol and its acetylated and modified forms in food and feed. EFSA J. (2017) 15:e04718. doi: 10.2903/j.efsa.2017.4718 32625635 PMC7010102

[30] EFSA. Appropriateness to set a group health based guidance value for T2 and HT2 toxin and its modified forms. EFS2. (2017) 15. doi: 10.2903/j.efsa.2017.4655 PMC7010130 32625252

[31] EFSA. Scientific opinion on the risks for public health related to the presence of zearalenone in food. EFS2. (2011) 9:2197. doi: 10.2903/j.efsa.2011.2197

[32] EFSA. Dietary exposure assessment to Alternaria toxins in the European population. EFS2. (2016) 14:e04654. doi: 10.2903/j.efsa.2016.4654 PMC13088870 42006026

[33] González-Jartín JM Rodríguez-Cañás I Alfonso A Sainz MJ Vieytes MR Gomes A . Multianalyte method for the determination of regulated, emerging and modified mycotoxins in milk: QuEChERS extraction followed by UHPLC-MS/MS analysis. Food Chem. (2021) 356:129647. doi: 10.1016/j.foodchem.2021.129647, 33813202

[34] Hamed AM Abdel-Hamid M Gámiz-Gracia L García-Campaña AM Arroyo-Manzanares N. Determination of aflatoxins in plant-based milk and dairy products by dispersive liquid-liquid microextraction and high-performance liquid chromatography with fluorescence detection. Anal Lett. (2019) 52:363–72. doi: 10.1080/00032719.2018.1467434

[35] Miró-Abella E Herrero P Canela N Arola L Borrull F Ras R . Determination of mycotoxins in plant-based beverages using QuEChERS and liquid chromatography-tandem mass spectrometry. Food Chem. (2017) 229:366–72. doi: 10.1016/j.foodchem.2017.02.078, 28372187

[36] Rodríguez-Cañás I González-Jartín JM Alfonso A Alvariño R Vieytes MR Botana LM. Application of a multi-toxin detect method to analyze mycotoxins occurrence in plant-based beverages. Food Chem. (2024) 434:137427. doi: 10.1016/j.foodchem.2023.137427, 37708575

[37] Juan C Mañes J Juan-García A Moltó JC. Multimycotoxin analysis in oat, rice, almond and soy beverages by liquid chromatography-tandem mass spectrometry. Appl Sci. (2022) 12. doi: 10.3390/app12083942

[38] Pavlenko R Berzina Z Reinholds I Bartkiene E Bartkevics V. An occurrence study of mycotoxins in plant-based beverages using liquid chromatography-mass spectrometry. Toxins. (2024) 16. doi: 10.3390/toxins16010053, 38251269 PMC10821093

[39] Kumari A Tirkey NN. Tenuazonic acid: a potent mycotoxin In: Singh K Srivastava N, editors. Recent trends in human and animal mycology. Singapore: Springer Singapore (2019). 203–11.

[40] Rahmani A Jinap S Soleimany F. Qualitative and quantitative analysis of mycotoxins. Compr Rev Food Sci Food Saf. (2009) 8:202–51. doi: 10.1111/j.1541-4337.2009.00079.x, 33467794

[41] Arroyo-Manzanares N Hamed AM García-Campaña AM Gámiz-Gracia L. Plant-based milks: unexplored source of emerging mycotoxins. A proposal for the control of enniatins and beauvericin using UHPLC-MS/MS. Food Addit Contam. (2019) 12:296–302. doi: 10.1080/19393210.2019.1663276, 31791225

[42] Mandel J. The statistical analysis of experimental data. New York, USA: Dover Publications (1964).

[43] Asam S Rychlik M. Potential health hazards due to the occurrence of the mycotoxin tenuazonic acid in infant food. Eur Food Res Technol. (2013) 236:491–7. doi: 10.1007/s00217-012-1901-x

[44] Arranz I Stroka J Neugebauer M. Determination of aflatoxin B 1 in tiger nut-based soft drinks. Food Addit Contam. (2006) 23:305–8. doi: 10.1080/02652030500415652, 16517532

[45] Lanzanova C Giorni P Bulla G Locatelli S Montanari M Alberti I . Investigation on the presence of mycotoxins in seed hemp varieties. Food Addit Contam. (2024) 41:400–9. doi: 10.1080/19440049.2024.2311850, 38408274

[46] EFSA. Scientific opinion on the risks to human and animal health related to the presence of beauvericin and enniatins in food and feed. EFS2. (2014) 12:3802. doi: 10.2903/j.efsa.2014.3802

[47] Moss R Barker S Falkeisen A Gorman M Knowles S McSweeney MB. An investigation into consumer perception and attitudes towards plant-based alternatives to milk. Food Res Int. (2022) 159:111648. doi: 10.1016/j.foodres.2022.111648, 35940773

[48] Redan BW Zuklic J Hryshko J Boyer M Wan J Sandhu A . Analysis of eight types of plant-based Milk alternatives from the United States market for target minerals and trace elements. J Food Compost Anal. (2023) 122:105457. doi: 10.1016/j.jfca.2023.105457, 37533790 PMC10392789

[49] German Federal Institute for Risk Assessment (2014). Arsenic in rice and rice products; BfR Opinion No. 018/2015 of 24 June 2014. Bundesbehörden und Einrichtungen im Geschäftsbereich des Bundesministeriums für Ernährung und Landwirtschaft (Berlin: BMEL).

[50] German Federal Institute for Risk Assessment (2007). Isolated isoflavones are not without risk; Updated BfR Expert Opinion No. 039/2007 of 3. April 2007. Bundesbehörden und Einrichtungen im Geschäftsbereich des Bundesministeriums für Ernährung und Landwirtschaft (Berlin: BMEL).

[51] Giugliano R Musolino N Ciccotelli V Ferraris C Savio V Vivaldi B . Soy, Rice and oat drinks: investigating chemical and biological safety in plant-based Milk alternatives. Nutrients. (2023) 15:2258. doi: 10.3390/nu15102258, 37242141 PMC10221834

