# Supplementary material for: Development of a high-throughput UHPLC–MS/MS multi-method for the analysis of mycotoxins in plant-based milk alternatives as the basis for risk assessment
Source: Front Nutr. 2026 Jan 15;12:1709925. doi: 10.3389/fnut.2025.1709925 (PMC12853641; doi:10.3389/fnut.2025.1709925)

Table SI1: Standardized spiking scheme of internal standards for all analytes for a sample amount of 10 mL of plant-based milk alternative

| Analyte | Internal Standard | Volume IS [µL] | Concentration IS [µg/mL] | Spike Level [µg/L] |
| --- | --- | --- | --- | --- |
| AOH | [^2^H_4_]-AOH | 50 | 1 | 5 |
| AME | [^2^H_4_]-AME | 10 | 0.1 | 0.1 |
| TeA | [^13^C_6_, ^15^N] | 25 | 2 | 5 |
| DON | [^13^C_15_]-DON | 10 | 10 | 10 |
| 3-AcDON | [^13^C_17_]-3AcDON | 10 | 1 | 1 |
| T2-T | [^13^C_4_]-T-2 | 50 | 0.1 | 0.5 |
| HT-2 | [^13^C_22_]-HT-2 | 10 | 1 | 1 |
| ENN A | [^15^N_3_]-ENN A1 | 20 | 1 | 2 |
| AFB1 | [^13^C_17_]-AFB1 | 10 | 0.1 | 0.1 |
| AFB2 | [^13^C_17_]-AFB2 | 20 | 0.1 | 0.1 |
| AFG1 | [^13^C_17_]-AFG2 | 20 | 0.1 | 0.2 |
| AFG2 | [^13^C_17_]-AFG2 | 20 | 0.1 | 0.2 |
| OTA | [^13^C_20_]-OTA | 50 | 0.1 | 0.5 |
| STC | [^13^C_18_]-STC | 10 | 0.1 | 0.1 |

Table SI2: Concentration levels for validation

| Analyte | LOD and LOQ | | | | Precision | | | Recovery | | | | |
| --- | --- | --- | --- | --- | --- | --- | --- | --- | --- | --- | --- | --- |
|  | Level 1  [µg/L] | Level 2  [µg/L] | Level 3  [µg/L] | Level 4  [µg/L] | Inter-injection  (n = 10) [µg/L] | Intra-day  (n = 3) [µg/L] | Inter-day  (n = 9) [µg/L] | Level 1  [µg/L] | Level 2  [µg/L] | Level 3  [µg/L] | Level 4  [µg/L] | Level 5  [µg/L] |
| AOH | 0.05 | 0.15 | 0.35 | 0.50 | 30 | 5 | 5 | 0.15 | 0.5 | 1 | 5 | 20 |
| AOH-3-G | 0.065 | 0.1 | 0.5 | 1.0 | 5 | 5 | 5 | 0.1 | 0.5 | 1 | 5 | 20 |
| AOH-3-S | 0.02 | 0.06 | 0.14 | 0.20 | 5 | 5 | 5 | 0.06 | 0.02 | 1 | 5 | 20 |
| AME | 0.002 | 0.006 | 0.014 | 0.020 | 2 | 5 | 5 | 0.006 | 0.02 | 0.2 | 1 | 5 |
| AME-3-S | 0.005 | 0.015 | 0.035 | 0.050 | 0.5 | 1 | 1 | 0.015 | 0.05 | 0.3 | 1 | 5 |
| TeA | 0.05 | 0.15 | 0.35 | 0.50 | 200 | 20 | 20 | 0.15 | 0.5 | 5 | 50 | 200 |
| ATX I | 0.1 | 0.3 | 0.7 | 1.0 | 150 | 10 | 10 | 0.3 | 1 | 5 | 10 | 20 |
| ALTP | 0.3 | 0.9 | 2.1 | 3.0 | 300 | 5 | 5 | 2.1 | 3 | 5 | 10 | 20 |
| TEN | 0.02 | 0.06 | 0.14 | 0.20 | 20 | 5 | 5 | 0.06 | 0.14 | 0.2 | 1 | 5 |
| DON | 0.3 | 0.9 | 2.1 | 3.0 | 100 | 50 | 50 | 0.9 | 3.0 | 10 | 50 | 200 |
| DON-3-G | 2 | 6 | 14 | 20 | 300 | 20 | 20 | 6 | 14 | 20 | 50 | 100 |
| 3-AcDON | 0.2 | 0.6 | 1.4 | 2.0 | 10 | 5 | 5 | 0.6 | 2.0 | 5.0 | 20 | 50 |
| NIV | 2 | 6 | 14 | 20 | 500 | 50 | 50 | 6 | 14 | 20 | 50 | 200 |
| HT-2 | 0.1 | 0.3 | 0.7 | 1.0 | 20 | 5 | 5 | 0.3 | 1 | 5 | 20 | 50 |
| Fus X | 0.4 | 1.2 | 2.8 | 4.0 | 50 | 50 | 50 | 1.2 | 4 | 10 | 30 | 50 |
| T-2 | 0.02 | 0.06 | 0.14 | 0.2 | 3 | 5 | 5 | 0.06 | 0.2 | 1 | 5 | 50 |
| ZEN | 0.02 | 0.06 | 0.14 | 0.20 | 20 | 5 | 5 | 0.06 | 0.2 | 1 | 5 | 50 |
| ENN A | 0.002 | 0.006 | 0.014 | 0.020 | 0.4 | 5 | 5 | 0.006 | 0.02 | 0.1 | 1 | 10 |
| ENN A1 | 0.002 | 0.006 | 0.014 | 0.020 | 0.4 | 5 | 5 | 0.006 | 0.02 | 0.1 | 1 | 10 |
| ENN B | 0.002 | 0.006 | 0.014 | 0.020 | 0.4 | 5 | 5 | 0.006 | 0.02 | 0.1 | 1 | 10 |
| ENN B1 | 0.002 | 0.006 | 0.014 | 0.020 | 0.4 | 5 | 5 | 0.006 | 0.02 | 0.1 | 1 | 10 |
| BEA | 0.002* | 0.005 | 0.01 | 0.02 | 0.4 | 5 | 5 | 0.0068 | 0.021 | 0.1 | 1 | 10 |
| OTA | 0.1 | 0.3 | 0.7 | 1.0 | 5 | 5 | 5 | 0.3 | 0.5 | 0.7 | 5 | 10 |
| STC | 0.0025 | 0.0075 | 0.0175 | 0.025 | 2 | 1 | 1 | 0.0075 | 0.025 | 0.1 | 1 | 5 |
| AFB1 | 0.0075 | 0.0225 | 0.0525 | 0.0750 | 2 | 0.5 | 0.5 | 0.0225 | 0.075 | 0.5 | 1 | 5 |
| AFB2 | 0.0075 | 0.0225 | 0.0525 | 0.0750 | 2 | 0.5 | 0.5 | 0.0225 | 0.075 | 0.5 | 1 | 2 |
| AFG1 | 0.01 | 0.03 | 0.07 | 0.1 | 2 | 0.5 | 0.5 | 0.03 | 0.1 | 0.5 | 1 | 5 |
| AFG2 | 0.05 | 0.15 | 0.35 | 0.50 | 2 | 0.5 | 0.5 | 0.15 | 0.35 | 0.5 | 1 | 2 |

* BEA was present naturally at a concentration near the LOD in the blank matrix. The amount that served for level 1 was quantified via standard addition.

Table SI3: Mean concentrations of all mycotoxins in all matrices [µg/L]

| Sample No. | Type | Main ingredient | DON  [µg/L] | DON-3-G  [µg/L] | 3-AcDON  [µg/L] | T-2  [µg/L] | HT-2  [µg/L] | ZEN  [µg/L] | ENN A1  [µg/L] | ENN B1  [µg/L] | ENN A  [µg/L] | ENN B  [µg/L] | BEA  [µg/L] | TeA  [µg/L] | AOH  [µg/L] | AOH-3-G  [µg/L] | AOH-3-S  [µg/L] | AME  [µg/L] | AME-3-S  [µg/L] | TEN  [µg/L] | OTA  [µg/L] | STC  [µg/L] | AFB1  [µg/L] | AFB2  [µg/L] | AFG1  [µg/L] |
| --- | --- | --- | --- | --- | --- | --- | --- | --- | --- | --- | --- | --- | --- | --- | --- | --- | --- | --- | --- | --- | --- | --- | --- | --- | --- |
| 1 | Based on oat | 9.8% oat | 0.7057 | n.d. | n.d. | 0.3589 | 1.0869 | n.d. | 0.0392 | 0.1055 | n.d. | 1.5813 | 0.0532 | 0.2712 | n.d. | n.d. | n.d. | <LoQ | n.d. | <LoQ | n.d. | 0.0155 | n.d. | n.d. | n.d. |
| 2 | Based on oat | 8.7% oat | 0.7868 | n.d. | n.d. | 0.3620 | 0.5765 | n.d. | 0.0527 | 0.1283 | n.d. | 2.3735 | 0.0532 | 7.2049 | n.d. | n.d. | n.d. | <LoQ | n.d. | 0.1476 | <LoQ | 0.0137 | n.d. | n.d. | n.d. |
| 3 | Based on oat | 8.7% oat | 0.7003 | n.d. | n.d. | 0.1746 | 0.3792 | n.d. | 0.0297 | 0.0843 | n.d. | 1.5320 | 0.0495 | 0.4941 | n.d. | n.d. | n.d. | n.d. | n.d. | <LoQ | n.d. | <LoQ | n.d. | n.d. | n.d. |
| 4 | Based on oat | 12% whole grain oat | 3.8411 | <LoQ | <LoQ | 0.2375 | 0.6844 | n.d. | 0.0749 | 0.1983 | n.d. | 5.8865 | 0.2350 | 3.3762 | n.d. | n.d. | n.d. | <LoQ | n.d. | 0.1452 | n.d. | n.d. | n.d. | n.d. | n.d. |
| 5 | Based on oat | 12% whole grain oat | 1.0257 | n.d. | n.d. | 0.1689 | 0.5937 | n.d. | 0.1044 | 0.2478 | n.d. | n.d. | 0.0474 | 1.1410 | n.d. | n.d. | n.d. | n.d. | n.d. | 0.1150 | n.d. | n.d. | n.d. | n.d. | n.d. |
| 6 | Based on oat | 7.8% oat | <LoQ | n.d. | n.d. | 0.1518 | 0.3065 | n.d. | 0.1068 | 0.2273 | n.d. | 1.0787 | 0.0467 | 0.2092 | n.d. | n.d. | n.d. | n.d. | n.d. | n.d. | n.d. | 0.0128 | n.d. | n.d. | <LoQ |
| 7 | Based on oat | 10% oat | n.d. | n.d. | n.d. | 0.2255 | 0.5157 | n.d. | 0.0261 | 0.1480 | <LoQ | 0.3647 | 0.0360 | n.d. | n.d. | n.d. | n.d. | n.d. | n.d. | 0.0950 | n.d. | 0.0070 | n.d. | n.d. | n.d. |
| 8 | Based on oat | 11.3% whole grain oat | <LoQ | n.d. | n.d. | 0.1791 | 0.3453 | n.d. | 0.0566 | 0.3276 | n.d. | 1.4615 | 0.0649 | 1.6485 | n.d. | n.d. | n.d. | <LoQ | n.d. | 0.1199 | n.d. | n.d. | n.d. | n.d. | n.d. |
| 9 | Based on oat | 11% whole grain oat | <LoQ | n.d. | n.d. | 0.0856 | 0.2139 | n.d. | 0.0289 | 0.1918 | n.d. | 0.9482 | 0.0493 | 0.6065 | n.d. | n.d. | n.d. | n.d. | n.d. | 0.1197 | n.d. | n.d. | n.d. | n.d. | n.d. |
| 10 | Based on oat | 9.2% oat | 1.4476 | n.d. | <LoQ | 0.2789 | 0.9797 | n.d. | 0.0861 | 0.4255 | 0.3646 | 1.5064 | 0.1030 | 0.8551 | n.d. | n.d. | n.d. | <LoQ | n.d. | 0.1152 | n.d. | n.d. | n.d. | n.d. | n.d. |
| 11 | Based on oat | 10% oat | 3.9037 | n.d. | <LoQ | 1.6738 | 7.9412 | <LoQ | 0.4180 | 2.4266 | <LoQ | 7.4033 | 0.0402 | 0.1160 | n.d. | n.d. | n.d. | n.d. | n.d. | n.d. | <LoQ | n.d. | n.d. | n.d. | n.d. |
| 12 | Based on oat | 11.9% oat | <LoQ | n.d. | n.d. | 0.0427 | <LoQ | n.d. | 0.0790 | 0.4944 | 0.0283 | 1.7513 | 0.1792 | 0.6614 | n.d. | n.d. | n.d. | n.d. | n.d. | 0.0874 | n.d. | n.d. | n.d. | n.d. | n.d. |
| 13 | Based on oat | 10% oat | <LoQ | n.d. | n.d. | 0.0636 | 0.2576 | n.d. | 0.0589 | 0.3204 | n.d. | 1.1304 | 0.2456 | 0.2534 | n.d. | n.d. | n.d. | n.d. | n.d. | 0.0647 | n.d. | n.d. | n.d. | n.d. | n.d. |
| 14 | Based on oat | 10% oat | <LoQ | n.d. | n.d. | 0.9201 | 2.0240 | n.d. | 0.0562 | 0.2775 | <LoQ | 0.6478 | 0.3502 | <LoQ | n.d. | n.d. | n.d. | n.d. | n.d. | n.d. | n.d. | 0.0060 | n.d. | n.d. | n.d. |
| 15 | Based on oat | 10% oat | 4.1026 | 7.3817 | 0.3707 | 1.1230 | 2.2725 | <LoQ | 0.2365 | 1.2085 | <LoQ | 3.1987 | 1.1159 | 1.7149 | n.d. | n.d. | n.d. | n.d. | n.d. | <LoQ | n.d. | n.d. | n.d. | n.d. | n.d. |
| 16 | Based on oat | 6% whole grain oat; 5.5% oat | 19.4140 | 11.5468 | <LoQ | 0.4616 | 1.1805 | 0.3998 | 1.4266 | 5.7829 | <LoQ | 17.4270 | 0.1952 | 1.0435 | n.d. | n.d. | n.d. | 0.0056 | n.d. | 0.1579 | n.d. | n.d. | n.d. | n.d. | n.d. |
| 17 | Based on oat | 10% oat | 3.8422 | 4.1231 | n.d. | 0.3291 | 0.7787 | <LoQ | 1.2528 | 4.8427 | n.d. | 13.1867 | 0.1989 | 0.9855 | n.d. | n.d. | n.d. | <LoQ | n.d. | 0.1109 | n.d. | n.d. | n.d. | n.d. | n.d. |
| 18 | Based on oat | 8.7% oat | <LoQ | n.d. | n.d. | 0.0761 | 0.2610 | n.d. | 0.0161 | 0.0726 | n.d. | 0.3460 | 0.0452 | 0.2633 | n.d. | n.d. | n.d. | n.d. | n.d. | <LoQ | n.d. | n.d. | n.d. | n.d. | n.d. |
| 19 | Based on oat | 11.5% oat | n.d. | n.d. | n.d. | 0.2070 | 0.3984 | n.d. | 0.0755 | 0.3325 | n.d. | 1.4626 | 0.0813 | 0.5318 | n.d. | n.d. | n.d. | n.d. | n.d. | <LoQ | n.d. | n.d. | n.d. | n.d. | n.d. |
| 20 | Based on oat | 15% whole grain oat | <LoQ | n.d. | n.d. | 0.1753 | 0.5584 | n.d. | 0.0891 | 0.5278 | n.d. | 2.2487 | 0.1007 | 1.2185 | n.d. | n.d. | n.d. | n.d. | n.d. | 0.0713 | n.d. | n.d. | n.d. | n.d. | n.d. |
| 21 | Based on oat | 11.5% whole grain oat | <LoQ | n.d. | n.d. | 0.0797 | <LoQ | n.d. | 0.0177 | 0.1200 | n.d. | 0.7299 | 0.0389 | 0.4845 | n.d. | n.d. | n.d. | n.d. | n.d. | <LoQ | n.d. | n.d. | n.d. | n.d. | n.d. |
| 22 | Based on oat | 11% oat | n.d. | n.d. | n.d. | 0.1012 | 0.4158 | n.d. | 0.0399 | 0.2227 | 1.0567 | 1.0335 | 0.0543 | 1.9974 | n.d. | n.d. | n.d. | n.d. | n.d. | 0.1655 | n.d. | 0.0174 | n.d. | n.d. | n.d. |
| 23 | Based on oat | 13% oat | 0.8018 | n.d. | n.d. | 0.3001 | 0.7886 | n.d. | 0.0702 | 0.3739 | 1.2647 | 1.5629 | 0.1403 | 0.3406 | n.d. | n.d. | n.d. | n.d. | n.d. | <LoQ | n.d. | n.d. | n.d. | n.d. | n.d. |
| 24 | Based on oat | 11% whole grain oat | <LoQ | n.d. | n.d. | 0.1071 | 0.2127 | n.d. | 0.0255 | 0.1687 | 1.2198 | 0.8199 | 0.0631 | 0.5067 | n.d. | n.d. | n.d. | n.d. | n.d. | 0.1018 | n.d. | n.d. | n.d. | n.d. | n.d. |
| 25 | Based on oat | 11% whole grain oat | n.d. | n.d. | n.d. | 0.1392 | 0.3437 | <LoQ | 0.0825 | 0.5670 | n.d. | 3.4093 | 0.0886 | 1.3858 | n.d. | n.d. | n.d. | n.d. | n.d. | 0.0884 | n.d. | 0.0782 | 0.0593 | n.d. | n.d. |
| 26 | Based on oat | 11.6% whole grain oat and oat | <LoQ | n.d. | n.d. | 0.3040 | 0.8681 | n.d. | 0.3252 | 1.5578 | n.d. | 5.8531 | 0.2024 | 0.9783 | n.d. | n.d. | n.d. | n.d. | n.d. | 0.1565 | n.d. | n.d. | n.d. | n.d. | n.d. |
| 27 | Based on oat | 10% oat | 2.6768 | 6.3323 | n.d. | 0.2891 | 0.5754 | n.d. | 0.0896 | 0.4196 | <LoQ | 1.1349 | 0.3754 | 1.1484 | n.d. | n.d. | n.d. | n.d. | n.d. | 0.1134 | n.d. | n.d. | n.d. | n.d. | n.d. |
| 28 | Based on oat | 14% whole grain oat | 0.9454 | n.d. | n.d. | 0.1255 | 0.2663 | n.d. | 0.0651 | 0.3541 | n.d. | 1.5636 | 0.4231 | 2.8846 | n.d. | n.d. | n.d. | n.d. | n.d. | 0.2567 | n.d. | n.d. | n.d. | n.d. | n.d. |
| 29 | Based on oat | 11.5% oat | <LoQ | n.d. | n.d. | 0.2274 | 0.5728 | n.d. | 0.0500 | 0.2355 | n.d. | 1.7551 | 0.1947 | 0.4540 | n.d. | n.d. | n.d. | n.d. | n.d. | <LoQ | n.d. | n.d. | n.d. | n.d. | n.d. |
| 30 | Based on oat | 11% oat | <LoQ | n.d. | n.d. | 0.1526 | 0.2359 | n.d. | 0.0172 | 0.0806 | n.d. | 0.7437 | 0.1241 | 0.8640 | n.d. | n.d. | n.d. | n.d. | n.d. | <LoQ | n.d. | n.d. | n.d. | n.d. | n.d. |
| 31 | Based on oat | 12% whole grain oat | <LoQ | n.d. | n.d. | 0.1773 | n.d. | n.d. | 0.1068 | 1.1269 | n.d. | 16.4822 | 0.4783 | 1.1167 | n.d. | n.d. | n.d. | n.d. | n.d. | 0.0677 | n.d. | 0.0109 | n.d. | n.d. | n.d. |
| 32 | Based on cereals | 14% rice | n.d. | n.d. | n.d. | n.d. | n.d. | n.d. | n.d. | n.d. | n.d. | n.d. | <LoQ | 2.8419 | n.d. | n.d. | n.d. | <LoQ | n.d. | <LoQ | n.d. | 0.0258 | n.d. | n.d. | n.d. |
| 33 | Based on cereals | 13% rice | n.d. | n.d. | n.d. | n.d. | n.d. | n.d. | n.d. | n.d. | n.d. | n.d. | <LoQ | 0.9590 | n.d. | n.d. | n.d. | 0.0123 | n.d. | <LoQ | n.d. | n.d. | n.d. | n.d. | n.d. |
| 34 | Based on cereals | 14% rice | n.d. | n.d. | n.d. | <LoQ | n.d. | <LoQ | n.d. | n.d. | n.d. | n.d. | 0.0059 | 0.8193 | n.d. | n.d. | n.d. | 0.0067 | n.d. | n.d. | n.d. | <LoQ | <LoQ | n.d. | n.d. |
| 35 | Based on cereals | 13% rice | n.d. | n.d. | n.d. | n.d. | n.d. | <LoQ | n.d. | n.d. | n.d. | 0.0036 | 0.0057 | 1.7572 | n.d. | n.d. | n.d. | 0.0484 | n.d. | <LoQ | n.d. | 0.0182 | 0.0235 | <LoQ | n.d. |
| 36 | Based on cereals | 13% rice | n.d. | n.d. | n.d. | n.d. | n.d. | <LoQ | n.d. | n.d. | n.d. | n.d. | 0.0061 | 0.6299 | n.d. | n.d. | n.d. | <LoQ | n.d. | <LoQ | n.d. | <LoQ | <LoQ | n.d. | n.d. |
| 37 | Based on cereals | 17% whole grain rice | n.d. | n.d. | n.d. | 0.0666 | n.d. | <LoQ | n.d. | n.d. | n.d. | 0.0039 | 0.0303 | 22.6103 | 0.3380 | n.d. | n.d. | 0.0405 | <LoQ | 0.2162 | n.d. | 0.0147 | n.d. | n.d. | n.d. |
| 38 | Based on cereals | 17% rice; 0.54% barley | n.d. | n.d. | n.d. | n.d. | n.d. | n.d. | <LoQ | 0.0070 | n.d. | 0.0422 | 0.0068 | 1.7752 | n.d. | n.d. | n.d. | n.d. | n.d. | 0.0685 | n.d. | n.d. | n.d. | n.d. | n.d. |
| 39 | Based on cereals | 17% rice; 3% almond | n.d. | n.d. | n.d. | n.d. | n.d. | n.d. | n.d. | n.d. | n.d. | n.d. | <LoQ | 8.7380 | n.d. | n.d. | n.d. | 0.0303 | 0.0211 | 0.0906 | <LoQ | 0.0200 | 0.0337 | <LoQ | n.d. |
| 40 | Based on cereals | 17% whole grain rice | <LoQ | n.d. | n.d. | <LoQ | n.d. | <LoQ | n.d. | n.d. | n.d. | n.d. | <LoQ | 14.1718 | n.d. | n.d. | n.d. | 0.0221 | n.d. | 0.0846 | n.d. | 0.0383 | n.d. | n.d. | n.d. |
| 41 | Based on cereals | 16% spelt | <LoQ | n.d. | n.d. | <LoQ | <LoQ | n.d. | 0.0116 | 0.0408 | n.d. | 0.2548 | 0.0102 | 5.2597 | n.d. | n.d. | n.d. | n.d. | n.d. | 0.1573 | n.d. | 0.0263 | n.d. | n.d. | n.d. |
| 42 | Based on cereals | 11% whole grain spelt | 1.7108 | n.d. | n.d. | n.d. | n.d. | n.d. | 0.0324 | 0.2126 | <LoQ | 0.8940 | n.d. | 1.0572 | n.d. | n.d. | n.d. | <LoQ | n.d. | 0.0832 | n.d. | n.d. | n.d. | n.d. | n.d. |
| 43 | Based on cereals | 11% whole grain spelt | 2.9665 | n.d. | n.d. | n.d. | 0.2082 | n.d. | 0.1173 | 0.6789 | n.d. | 3.2480 | 0.0082 | 1.1918 | n.d. | n.d. | n.d. | n.d. | n.d. | <LoQ | n.d. | n.d. | n.d. | n.d. | n.d. |
| 44 | Based on cereals | 11% whole grain spelt | 1.2357 | n.d. | n.d. | 0.0446 | 0.4719 | n.d. | 0.0925 | 0.7067 | 0.0114 | 3.4645 | <LoQ | 1.3509 | n.d. | n.d. | n.d. | <LoQ | n.d. | <LoQ | n.d. | n.d. | n.d. | n.d. | n.d. |
| 45 | Based on cereals | 17% whole grain spelt | <LoQ | n.d. | n.d. | n.d. | <LoQ | n.d. | 0.0095 | 0.0578 | n.d. | 0.1497 | 0.0279 | 1.6371 | n.d. | n.d. | n.d. | <LoQ | n.d. | 0.0982 | n.d. | n.d. | n.d. | n.d. | n.d. |
| 46 | Based on cereals | 15% millet | <LoQ | n.d. | n.d. | 0.1168 | <LoQ | n.d. | 0.0044 | 0.0193 | n.d. | 0.2056 | 0.0065 | 50.4629 | n.d. | n.d. | n.d. | 0.0223 | n.d. | 0.1156 | n.d. | n.d. | n.d. | n.d. | n.d. |
| 47 | Based on cereals | 16% millet | 0.8695 | n.d. | n.d. | <LoQ | n.d. | n.d. | 0.0054 | 0.0287 | n.d. | 0.0822 | 0.0109 | 62.0128 | 0.1596 | n.d. | n.d. | 0.0167 | n.d. | 0.2097 | n.d. | 0.0065 | 0.1042 | <LoQ | n.d. |
| 48 | Based on cereals | 16% millet | n.d. | n.d. | n.d. | <LoQ | n.d. | <LoQ | n.d. | n.d. | n.d. | n.d. | 0.0061 | 7.6021 | n.d. | n.d. | n.d. | 0.0106 | n.d. | 0.1133 | n.d. | 0.0111 | n.d. | n.d. | n.d. |
| 49 | Based on cereals | 12.5% buckwheat | n.d. | n.d. | n.d. | <LoQ | n.d. | <LoQ | <LoQ | 0.0411 | n.d. | n.d. | <LoQ | 0.4639 | 0.4918 | 0.6110 | n.d. | <LoQ | n.d. | <LoQ | n.d. | <LoQ | n.d. | n.d. | n.d. |
| 50 | Based on cereals | 12.5% buckwheat | 0.6927 | n.d. | n.d. | 0.0520 | 0.2736 | 0.0648 | 0.0128 | 0.0625 | n.d. | 0.1613 | 0.0064 | 8.5869 | 2.2176 | 0.3942 | n.d. | 0.0458 | 0.0170 | 0.0821 | n.d. | 0.4173 | 0.0763 | <LoQ | <LoQ |
| 51 | Based on cereals | 8% buckwheat; 7% rice | n.d. | n.d. | n.d. | n.d. | n.d. | n.d. | n.d. | n.d. | n.d. | 0.0070 | 0.0110 | 6.5390 | 1.2028 | 0.3310 | n.d. | 0.0118 | n.d. | 0.1087 | n.d. | 0.0190 | n.d. | n.d. | n.d. |
| 52 | Based on cereals | 7.2% oat; 2.3% almond | <LoQ | n.d. | n.d. | <LoQ | <LoQ | n.d. | 0.0209 | 0.0527 | n.d. | 1.3752 | 0.0688 | 0.8297 | n.d. | n.d. | n.d. | n.d. | n.d. | 0.0520 | n.d. | n.d. | n.d. | n.d. | n.d. |
| 53 | Based on cereals | 3.2% oat; 1.7% almond | n.d. | n.d. | n.d. | 0.0713 | 0.1702 | n.d. | 0.0442 | 0.1043 | n.d. | 0.4306 | 0.0147 | 1.8087 | n.d. | n.d. | n.d. | <LoQ | n.d. | n.d. | n.d. | 0.0089 | 0.0335 | <LoQ | n.d. |
| 54 | Based on cereals | 5.5% soy; 5.3% rice | n.d. | n.d. | n.d. | n.d. | n.d. | n.d. | <LoQ | 0.0184 | <LoQ | 0.0452 | <LoQ | 0.6250 | n.d. | n.d. | n.d. | n.d. | n.d. | 0.0687 | n.d. | n.d. | n.d. | n.d. | n.d. |
| 55 | Based on cereals | 4.5% sunflower seed; 3.7% rice | n.d. | n.d. | n.d. | n.d. | n.d. | n.d. | <LoQ | <LoQ | n.d. | 0.0176 | <LoQ | 12.5709 | n.d. | n.d. | n.d. | 0.0119 | n.d. | 0.1622 | n.d. | 0.0127 | n.d. | n.d. | n.d. |
| 56 | Based on nuts | 2.3% almond | n.d. | n.d. | n.d. | n.d. | n.d. | <LoQ | <LoQ | <LoQ | n.d. | 0.0217 | <LoQ | 5.6570 | n.d. | n.d. | n.d. | 0.0019 | n.d. | 0.0485 | n.d. | <LoQ | 0.0162 | n.d. | n.d. |
| 57 | Based on nuts | 2.5% almond | n.d. | n.d. | n.d. | n.d. | n.d. | n.d. | n.d. | n.d. | n.d. | n.d. | n.d. | 0.5445 | <LoQ | n.d. | <LoQ | 0.0178 | <LoQ | n.d. | n.d. | n.d. | n.d. | n.d. | n.d. |
| 58 | Based on nuts | 2.3% almond | n.d. | n.d. | n.d. | n.d. | n.d. | <LoQ | <LoQ | <LoQ | n.d. | 0.0042 | n.d. | 4.3222 | n.d. | <LoQ | n.d. | 0.0059 | <LoQ | 0.0550 | n.d. | 0.0110 | 0.0916 | <LoQ | <LoQ |
| 59 | Based on nuts | 2.3% almond | n.d. | n.d. | n.d. | n.d. | n.d. | <LoQ | <LoQ | <LoQ | n.d. | 0.0039 | n.d. | 8.2066 | n.d. | n.d. | n.d. | 0.0137 | <LoQ | 0.0615 | n.d. | 0.0086 | 0.0847 | <LoQ | <LoQ |
| 60 | Based on nuts | 2.3% almond | n.d. | n.d. | n.d. | n.d. | n.d. | <LoQ | 0.0047 | 0.0070 | n.d. | 0.0173 | <LoQ | 4.3146 | n.d. | <LoQ | n.d. | 0.0094 | <LoQ | 0.0788 | <LoQ | 0.0054 | <LoQ | n.d. | <LoQ |
| 61 | Based on nuts | 7% almond | n.d. | n.d. | n.d. | n.d. | n.d. | <LoQ | <LoQ | 0.0040 | n.d. | 0.0070 | n.d. | 3.3308 | n.d. | n.d. | n.d. | <LoQ | n.d. | 0.0703 | n.d. | <LoQ | <LoQ | n.d. | n.d. |
| 62 | Based on nuts | 2.3% almond | n.d. | n.d. | n.d. | n.d. | n.d. | n.d. | <LoQ | <LoQ | n.d. | <LoQ | n.d. | 1.2235 | n.d. | n.d. | n.d. | 0.0125 | <LoQ | 0.0885 | n.d. | n.d. | n.d. | n.d. | n.d. |
| 63 | Based on nuts | 2.3% almond | n.d. | n.d. | n.d. | n.d. | n.d. | n.d. | <LoQ | 0.0055 | 0.0349 | 0.0136 | n.d. | 0.1495 | n.d. | n.d. | n.d. | n.d. | n.d. | n.d. | n.d. | n.d. | n.d. | n.d. | n.d. |
| 64 | Based on nuts | 2.3% almond | n.d. | n.d. | n.d. | n.d. | n.d. | <LoQ | <LoQ | <LoQ | <LoQ | <LoQ | <LoQ | 9.5399 | n.d. | n.d. | n.d. | <LoQ | n.d. | <LoQ | n.d. | 0.0137 | n.d. | n.d. | n.d. |
| 65 | Based on nuts | 2.3% almond | n.d. | n.d. | n.d. | n.d. | n.d. | n.d. | <LoQ | <LoQ | 0.0064 | n.d. | <LoQ | 9.6274 | n.d. | n.d. | n.d. | <LoQ | n.d. | <LoQ | n.d. | 0.0168 | n.d. | n.d. | n.d. |
| 66 | Based on nuts | 4% almond | n.d. | n.d. | n.d. | n.d. | n.d. | n.d. | <LoQ | <LoQ | <LoQ | <LoQ | <LoQ | 1.2415 | 0.4168 | n.d. | <LoQ | 0.0417 | 0.0299 | <LoQ | n.d. | n.d. | <LoQ | n.d. | n.d. |
| 67 | Based on nuts | 3% almond | n.d. | n.d. | n.d. | n.d. | n.d. | <LoQ | <LoQ | <LoQ | 0.0488 | <LoQ | n.d. | 1.2768 | n.d. | n.d. | n.d. | <LoQ | n.d. | <LoQ | n.d. | <LoQ | <LoQ | n.d. | n.d. |
| 68 | Based on nuts | 6% almond | n.d. | n.d. | n.d. | n.d. | n.d. | n.d. | <LoQ | <LoQ | n.d. | <LoQ | n.d. | 0.5761 | n.d. | n.d. | n.d. | <LoQ | n.d. | n.d. | n.d. | n.d. | n.d. | n.d. | n.d. |
| 69 | Based on nuts | 2.3% almond | n.d. | n.d. | n.d. | n.d. | n.d. | n.d. | <LoQ | n.d. | n.d. | n.d. | n.d. | 0.4540 | n.d. | n.d. | n.d. | n.d. | n.d. | n.d. | n.d. | n.d. | n.d. | n.d. | n.d. |
| 70 | Based on nuts | 5% almond; 1.5% chia seed & 0.4% chia oil | n.d. | n.d. | n.d. | n.d. | n.d. | n.d. | <LoQ | <LoQ | n.d. | 0.0052 | <LoQ | 3.3717 | n.d. | n.d. | n.d. | <LoQ | n.d. | 0.0618 | <LoQ | 0.0069 | 0.0397 | <LoQ | <LoQ |
| 71 | Based on nuts | 6.6% cashew | n.d. | n.d. | n.d. | n.d. | n.d. | n.d. | n.d. | n.d. | n.d. | n.d. | n.d. | <LoQ | n.d. | n.d. | n.d. | n.d. | n.d. | n.d. | n.d. | n.d. | n.d. | n.d. | n.d. |
| 72 | Based on nuts | 3.1% cashew | n.d. | n.d. | n.d. | n.d. | n.d. | n.d. | n.d. | n.d. | n.d. | n.d. | n.d. | 2.8053 | n.d. | n.d. | n.d. | <LoQ | n.d. | <LoQ | n.d. | n.d. | <LoQ | n.d. | n.d. |
| 73 | Based on nuts | 6.5% cashew | n.d. | n.d. | n.d. | n.d. | n.d. | n.d. | n.d. | n.d. | n.d. | n.d. | n.d. | n.d. | n.d. | n.d. | n.d. | n.d. | n.d. | n.d. | n.d. | n.d. | n.d. | n.d. | n.d. |
| 74 | Based on nuts | 6% cashew | n.d. | n.d. | n.d. | n.d. | n.d. | n.d. | n.d. | n.d. | n.d. | n.d. | n.d. | n.d. | n.d. | n.d. | n.d. | n.d. | n.d. | n.d. | n.d. | n.d. | n.d. | n.d. | n.d. |
| 75 | Based on nuts | 6.5% cashew | n.d. | n.d. | n.d. | n.d. | n.d. | n.d. | n.d. | n.d. | n.d. | n.d. | n.d. | n.d. | n.d. | n.d. | n.d. | n.d. | n.d. | n.d. | n.d. | n.d. | <LoQ | n.d. | n.d. |
| 76 | Based on nuts | 2.8% hazelnut | n.d. | n.d. | n.d. | 0.0389 | n.d. | 0.0551 | 2.3880 | 5.5793 | 0.7496 | 7.0964 | 0.0161 | 85.9370 | 4.3971 | n.d. | 0.0846 | 0.2674 | 0.0978 | 0.1545 | <LoQ | 0.0069 | 0.0145 | n.d. | n.d. |
| 77 | Based on nuts | 5% hazelnut | n.d. | n.d. | n.d. | n.d. | n.d. | <LoQ | 5.6959 | 13.8211 | n.d. | 18.4194 | 0.0068 | 72.5498 | 1.8250 | n.d. | <LoQ | 0.1729 | <LoQ | 0.0783 | n.d. | n.d. | n.d. | n.d. | n.d. |
| 78 | Based on nuts | 5.5% hazelnut | n.d. | n.d. | n.d. | n.d. | n.d. | <LoQ | 0.0056 | 0.0101 | 0.0099 | 0.0119 | <LoQ | <LoQ | 0.1280 | n.d. | n.d. | 0.0090 | n.d. | n.d. | n.d. | n.d. | <LoQ | n.d. | n.d. |
| 79 | Based on nuts | 3.8% wal nut | n.d. | n.d. | n.d. | 0.0744 | n.d. | <LoQ | 0.4436 | 1.0179 | n.d. | 1.0167 | 0.1024 | 1.9154 | 2.4887 | n.d. | n.d. | 0.1262 | <LoQ | 0.0627 | <LoQ | 0.0067 | n.d. | n.d. | n.d. |
| 80 | Based on nuts | cereals (spelt. rice. oat); 2% hazelnut; 1% almond; 0.5% macadamia | 4.0713 | n.d. | n.d. | 0.0524 | 0.1889 | <LoQ | 0.1456 | 0.6206 | n.d. | 1.6935 | 0.0081 | 6.9036 | 0.6931 | n.d. | n.d. | 0.0665 | n.d. | 0.1043 | n.d. | 0.2840 | n.d. | n.d. | n.d. |
| 81 | Based on nuts | 12% tiger nut | n.d. | n.d. | n.d. | n.d. | n.d. | n.d. | n.d. | n.d. | n.d. | n.d. | 0.0139 | 43.8755 | n.d. | n.d. | n.d. | 0.0060 | n.d. | n.d. | <LoQ | 0.0122 | 0.3217 | 0.0439 | 0.1111 |
| 82 | Based on nuts | 20.8% tigernut | n.d. | n.d. | n.d. | n.d. | n.d. | n.d. | n.d. | n.d. | n.d. | 0.0102 | 0.0127 | 35.8125 | <LoQ | n.d. | 0.0682 | 0.1081 | 0.0482 | 0.0682 | n.d. | 0.0143 | 0.3671 | 0.0484 | 0.0531 |
| 83 | Based on nuts | 12% tigernut | n.d. | n.d. | n.d. | n.d. | n.d. | n.d. | n.d. | n.d. | n.d. | 0.0082 | 0.0153 | n.d. | n.d. | n.d. | n.d. | 0.0187 | n.d. | n.d. | n.d. | 0.0196 | 0.6744 | 0.0633 | 0.1837 |
| 84 | Based on nuts | 12.5% tigernut | n.d. | n.d. | n.d. | n.d. | n.d. | n.d. | n.d. | n.d. | n.d. | 0.0093 | 0.0203 | n.d. | <LoQ | n.d. | n.d. | 0.0322 | n.d. | n.d. | n.d. | 0.0168 | 0.5010 | 0.0445 | 0.1170 |
| 85 | Based on nuts | 7.5% chestnut | n.d. | n.d. | n.d. | n.d. | n.d. | <LoQ | 0.0157 | 0.0305 | n.d. | 0.0455 | <LoQ | 0.6395 | 0.8414 | n.d. | n.d. | 0.0798 | <LoQ | 0.1218 | n.d. | n.d. | n.d. | n.d. | n.d. |
| 86 | Based on nuts | 3.3% pistachio | n.d. | n.d. | n.d. | n.d. | n.d. | n.d. | 0.0042 | 0.0041 | n.d. | 0.0081 | <LoQ | 4.4636 | n.d. | n.d. | n.d. | 0.0059 | n.d. | 0.0540 | <LoQ | n.d. | n.d. | n.d. | n.d. |
| 87 | Based on soy, peas and hemp | 8% soy | n.d. | n.d. | n.d. | n.d. | n.d. | n.d. | <LoQ | <LoQ | n.d. | n.d. | 0.0068 | 0.6985 | n.d. | n.d. | n.d. | n.d. | n.d. | <LoQ | n.d. | n.d. | n.d. | n.d. | n.d. |
| 88 | Based on soy, peas and hemp | 8% soy | n.d. | n.d. | n.d. | n.d. | n.d. | n.d. | 0.0042 | 0.0165 | <LoQ | 0.0357 | <LoQ | <LoQ | n.d. | n.d. | n.d. | n.d. | n.d. | n.d. | n.d. | n.d. | n.d. | n.d. | n.d. |
| 89 | Based on soy, peas and hemp | 8% soy | <LoQ | n.d. | n.d. | <LoQ | n.d. | <LoQ | <LoQ | 0.0182 | n.d. | 0.0898 | 0.0079 | 0.4459 | n.d. | n.d. | n.d. | n.d. | n.d. | n.d. | n.d. | n.d. | n.d. | n.d. | n.d. |
| 90 | Based on soy, peas and hemp | 9% soy | n.d. | n.d. | n.d. | n.d. | n.d. | n.d. | 0.0083 | 0.0364 | 0.0087 | 0.0837 | <LoQ | 0.1307 | n.d. | n.d. | n.d. | n.d. | n.d. | n.d. | n.d. | n.d. | n.d. | n.d. | n.d. |
| 91 | Based on soy, peas and hemp | 9.8% soy | <LoQ | n.d. | n.d. | 0.1213 | n.d. | n.d. | 0.0117 | 0.0528 | n.d. | 0.1714 | 0.0198 | 0.4644 | n.d. | n.d. | n.d. | n.d. | n.d. | 0.0668 | n.d. | n.d. | n.d. | n.d. | n.d. |
| 92 | Based on soy, peas and hemp | 7% soy | n.d. | n.d. | n.d. | n.d. | n.d. | n.d. | <LoQ | <LoQ | <LoQ | 0.0048 | 0.0154 | n.d. | n.d. | n.d. | n.d. | n.d. | n.d. | n.d. | <LoQ | n.d. | n.d. | n.d. | n.d. |
| 93 | Based on soy, peas and hemp | 8% soy | n.d. | n.d. | n.d. | n.d. | n.d. | n.d. | 0.0093 | 0.0289 | 0.0071 | 0.0621 | <LoQ | 0.2580 | n.d. | n.d. | n.d. | n.d. | n.d. | <LoQ | n.d. | n.d. | n.d. | n.d. | n.d. |
| 94 | Based on soy, peas and hemp | 9% pea | n.d. | n.d. | n.d. | n.d. | n.d. | <LoQ | <LoQ | 0.0076 | n.d. | 0.0591 | n.d. | <LoQ | n.d. | n.d. | n.d. | <LoQ | n.d. | <LoQ | n.d. | n.d. | n.d. | n.d. | n.d. |
| 95 | Based on soy, peas and hemp | 9% pea | n.d. | n.d. | n.d. | n.d. | n.d. | n.d. | 0.0033 | 0.0188 | n.d. | 0.0834 | n.d. | <LoQ | n.d. | n.d. | n.d. | 0.0090 | n.d. | 0.0981 | n.d. | n.d. | n.d. | n.d. | n.d. |
| 96 | Based on soy, peas and hemp | 2.5% pea | n.d. | n.d. | n.d. | n.d. | n.d. | <LoQ | <LoQ | <LoQ | 0.0075 | 0.0099 | <LoQ | 0.1806 | n.d. | n.d. | n.d. | n.d. | n.d. | <LoQ | <LoQ | 0.0117 | n.d. | n.d. | n.d. |
| 97 | Based on soy, pea, and hemp | 3% pea | n.d. | n.d. | n.d. | n.d. | n.d. | n.d. | <LoQ | 0.0037 | 1.0826 | 0.0099 | <LoQ | <LoQ | n.d. | n.d. | n.d. | n.d. | n.d. | <LoQ | n.d. | n.d. | n.d. | n.d. | n.d. |
| 98 | Based on soy, peas and hemp | 3% hemp seeds; 1.3% hemp oil | n.d. | n.d. | n.d. | n.d. | n.d. | 0.3165 | 0.0243 | 0.0562 | n.d. | 0.2420 | <LoQ | 39.5378 | n.d. | n.d. | n.d. | 0.0268 | n.d. | 0.0807 | <LoQ | 0.0721 | n.d. | n.d. | n.d. |
| 99 | Based on soy, peas and hemp | 3% hemp seeds | 9.7295 | n.d. | <LoQ | 0.1809 | <LoQ | <LoQ | 0.0696 | 0.2112 | n.d. | 0.3413 | 0.0115 | 12.7899 | 0.1650 | n.d. | n.d. | 0.0310 | <LoQ | 0.2391 | n.d. | n.d. | n.d. | n.d. | n.d. |
| 100 | Based on soy, peas and hemp | 3% hemp seeds | <LoQ | n.d. | n.d. | n.d. | n.d. | 0.4114 | 0.0089 | 0.0324 | n.d. | 0.0847 | <LoQ | 0.4632 | n.d. | n.d. | n.d. | n.d. | n.d. | 0.0849 | n.d. | n.d. | n.d. | n.d. | n.d. |

Figure SI1: Mean concentrations of all mycotoxins in all matrices [µg/L]; Oat drinks (A), cereal drinks (B), nut drinks (C) und other drinks (D).


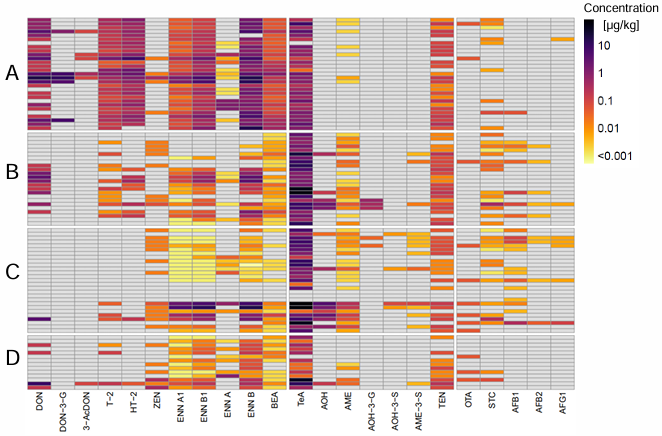

Supplement: Supplementary file 1 [file Supplementary_file_1.docx]
